# Iron oxide/silver-doped iron oxide nanoparticles: facile synthesis, characterization, antibacterial activity, genotoxicity and anticancer evaluation

**DOI:** 10.1038/s41598-025-14098-6

**Published:** 2025-08-12

**Authors:** Sara Abdelghany, Ashraf Elsayed, Hoda Kabary, Hosam Salaheldin

**Affiliations:** 1 Botany Department, Faculty of Science, Mansoura University, Mansoura, 35516 Egypt; 2 Agricultural Microbiology Department, National Research Center (NRC), Cairo, 12622 Egypt; 3 Biophysics Research Group, Department of Physics, Faculty of Science, Mansoura University, Mansoura, 35516 Egypt

**Keywords:** IONPs, Silver NPs doping, Antibacterial, Anticancer activity, Cell Cytotoxicity, Genotoxicity, Biotechnology, Microbiology, Nanoscience and technology

## Abstract

Iron oxide nanoparticles (IONPs) are extremely sought after due to their antibacterial, antioxidant, and anticancer properties. IONPs were synthesized from *Pseudomonas aeruginosa* kb1 extracellular supernatant extract. After 48 h at 37 °C in the precursor iron salt, the weak yellow culture supernatant turned yellowish-brown and brown-black, confirming IONP production. To make Ag-doped IONPs, sodium borohydride (NaBH_4_) reduced the silver nitrate (AgNO_3_) salt on the biosynthesized IONPs. SEM showed that the nanoparticles clustered and had a uniform size distribution and approximately spherical shape. EDX and XRD analysis validated the production of maghemite (γ-Fe_2_O_3_) and magnetite (Fe_3_O_4_) IONPs. Fourier transform infrared spectroscopy determined the surface functional groups of Ag-doped and IONPs. The antibacterial activity of Fe_3_O_4_ and Ag-doped Fe_3_O_4_ NPs against numerous harmful bacterial strains was much higher than that of Fe_2_O_3_. The normal retina cell line and human lung cancer cell line A549 were also tested for cytotoxicity using the MTT assay. Ag-doped Fe_3_O_4_ NPs were more cytotoxic than IONPs on A549 cells. Therefore, the biosynthesized Ag-doped Fe_3_O_4_ NPs, rather than IONPs, have potential applications as pharmaceutical and therapeutic products because they are safe, eco-friendly, and cost-effective.

## Introduction

Microbiological diseases or infections pose significant risks and economic burdens to human society. In 2017, the World Health Organization (WHO) published a report highlighting the urgent need to develop contemporary antimicrobial drugs that are essential for alleviating diseases and threats produced by microbes such as bacteria, viruses, fungi, and parasites to promote global health^1^. The sustainable development goals (SDGs) era has commenced. Among the SDGs, health is prominently positioned. The health target (SDG 3) is comprehensive: ‘Assurance of good health and promotion of well-being for individuals of all ages’. Consequently, novel and effective methods for addressing resistance to existing drugs are urgently needed. Hence, nanotechnology presents novel opportunities for the synthesis of nanomaterials possessing promising anticancer and antibacterial properties to address these constraints. Nanotechnology is the scientific study of manipulating and fabricating nanoparticles. Nanoparticles are characterized by having at least one or two dimensions that are 100 nm or less. These particles have a distinct property that separates them from bulk materials. Compared with particles of the same composition, their small size facilitates a high surface-to-volume ratio, resulting in significant biochemical and catalytic activity. In the fields of medication delivery, medicinal sciences, gene delivery, chemical industries, optics, mechanics, catalysis, and others, nanoparticles have extensive applications^2^.

Iron oxide nanoparticles (IONPs) have drawn attention among metallic nanoparticles because of their biocompatibility, availability, and proven efficacy in a wide range of applications, including sensor production, drug delivery, cancer therapy, and biomedical treatments^3^. Furthermore, iron oxide crystallites are present in several phases, including maghemite (γ-Fe_2_O_3_), hematite (α-Fe_2_O_3_), magnetite (Fe_3_O_4_), and goethite FeOH (OH). The most prevalent iron phases are hematite and magnetite, which are both natural and ecologically beneficial. They play an important role in the natural cycling of iron in the environment^4^. The fabrication of abiotic magnetite nanoparticles involves several chemical processes, including oxidative precipitation^5^, thermal decomposition^6^, microemulsion^7^, the sol-gel process^8^, and solvothermal^9^ procedures. Furthermore, the fabrication of nanoparticles requires an extra processing step to avoid particle aggregation, which further complicates the procedure. In contrast to chemical and physical approaches, the biological synthesis of nanoparticles using microorganisms or plant extracts yields nanoparticles with more precise size and shape control without the need for dangerous chemicals^10,11^. Biosynthesis can be efficiently carried out by employing various plants, bacteria, fungi, yeasts, and actinomycetes^12^. Hence, the process of nanoparticle biosynthesis plays a role in achieving sustainable development objectives, whereas the other physical and chemical approaches strictly rely on limited and costly resources for nanoparticle growth^13^.

The synthesis of NPs by microorganisms can be categorized into either intracellular or extracellular biosynthesis. Compared with those formed by intracellular synthesis, nanoparticles generated by extracellular synthesis are more easily purified and recovered^14,15^. In contrast, intracellular synthesis requires centrifugation followed by a sequence of ultrasonic cycles to disrupt the cells, therefore complicating the purifying process. The intracellular production of NPs involves the translocation of ions and chemicals into bacterial cells facilitated by enzymes^16,17^. Consequently, Abd, Mohsen^18^ reported that, compared with the extracellular method, the internal process may require more time to generate NPs.

As evidenced by the often uneven size and shape of magnetite crystals, these methods yield magnetite crystals that exhibit little to no control over the mineralization process. The development of biomimetic magnetite nanoparticles has led to advancements in the characterization of the proteins that are responsible for the generation of magnetosomes in magnetotactic bacteria (MTB)^19^. MTBs such as *Pseudomonas aeruginosa* (*P. aeruginosa*) and *Escherichia coli* (*E. coli*) are a broadly distributed collection of aquatic, Gram-negative, motile prokaryotes that exhibit morphologically, metabolically, and phylogenetically varied characteristics. They are commonly found in natural aquatic environments^20^. Since they are extensively distributed throughout several phyla within the bacterial domain, the term “magnetotactic bacteria” has no authentic taxonomic meaning^21^. MTBs share a unique magnetotactic behaviour known as magnetotaxis, characterized by passive alignment and movement along magnetic field lines. This behaviour is attributed to the presence of magnetosomes, which are intracellular crystals of nanometer-sized magnetite (Fe_3_O_4_) and/or greigite (Fe_3_S_4_) encased in a membrane bilayer^22^.

Adequate surface modification is required to make IONPs stable and enhance their surface activity, biocompatibility, and mechanical and physicochemical characteristics^23^. As previously reported^24,25^, doping is the most widely investigated strategy for modifying NPs to improve their biological capabilities. The doping approach is the most frequent surface modification method for attaching organic and inorganic compounds on the surface of IONPs. Furthermore, this approach prevents IONP oxidation and aggregation. Doping modification is also one of the most successful techniques for controlling and regulating how NPs interact with bacteria. The potential to offer the possibility for extra functionalization could improve their antibacterial and anticancer properties, making them appropriate choices for the biomedical field^26^.

Metal NPs, particularly silver nanoparticles (AgNPs), are highly desirable because of their potent antibacterial and anti-inflammatory properties. Atomic AgNPs have applications in several physical, biological, and pharmaceutical domains. For example, creams or ointments containing AgNPs are used to treat burns and wounds to prevent bacterial infection^27,28^. Ganesan, Jothi^29^ reported the synthesis of graphene oxide-copper oxide (GO-CuO) NPs because of their very efficient photocatalytic activity toward methylene blue and their lethal effects on human colon cancer cells. The antibacterial activity of phytomolecule-coated nickel oxide (NiO) NPs was examined by Khan, Shahid^30^. These findings revealed that the phytomolecule-coated NiO NPs were more effective against *S. aureus* (23 ± 0.77 mm) than against *E. coli* (18 ± 0.58 mm)^18^. In their study, Atacan, Güy^31^ provided the first documented example of the production of silver-doped metal oxide (MO, where M: Zn, Cu, Ni) NPs to investigate their antibacterial and catalytic properties. Silver-metal oxide NPs (Ag-MO NPs) have shown exceptional antibacterial and catalytic activities among MO NPs^31^.

The antibacterial efficacy of Ag-CuO NPs against *E. coli* and *S. aureus* bacteria has been demonstrated to be unparalleled, as evidenced by the highest observed inhibition zones. Compared with Ag-ZnO and Ag-NiO nanoparticles, Ag-CuO nanoparticles exhibited greater catalytic activity for 4-NA conversion^31^.

This work aims to study the synthesis of Fe_3_O_4_ and Fe_2_O_3_ NPs from magnetotactic (*P. aeruginosa*) bacterial extracts for various biological uses. Additionally, Fe_3_O_4_ and Fe_2_O_3_ are employed as precursors in the chemical reduction process to produce Ag-doped Fe_3_O_4_ and Ag-doped Fe_2_O_3_ NPs. The genotoxic and antibacterial activities of these NPs against a range of pathogens were then investigated. Further, the anticancer of the IONPs efficacy were evaluated against healthy retinal (RPE1) cell lines as well as in lung cancer (A549) and colon cancer (HCT116) cell lines.

## Materials and methods

### Materials

Ferrous sulfate heptahydrate (FeSO_4_.7H_2_O), ferric sulfate pentahydrate (Fe_2_(SO_4_)_3_.5H_2_O), silver nitrate (AgNO_3_), sodium borohydride (NaBH_4_), bacterial culture media and antibiotic solution were supplied by Merck (Germany). MTT (3-(4,5-dimethylthiazol-2-yl)−2,5-diphenyl tetrazolium bromide) and Comet assay chemicals were procured from Sigma‒Aldrich Company (Germany). All chemicals and solvents were of analytical grade and utilized without any additional purification. *Staphylococcus aureus* (ATCC 6538), methicillin-resistant *Staphylococcus aureus* (EMCC number 1353 t), *Escherichia coli* (ATCC 10536), *Klebsiella pneumoniae* (ATCC 10031), and *Salmonella typhi* (ATCC 25566) were obtained from the Microbiological Resources Centre (Cairo Mircen). The sources of the lung cancer cell line (A549), colon cancer cell line (HCT116), and normal retina cell line (RPE1) were ATCC USA.

### Culture collection and Preparation

The magnetotactic bacterium *Pseudomonas aeruginosa* kb1 (KT962901) was obtained from the microbial culture collection of the Agricultural Microbiology Department, National Research Centre, Cairo, Egypt. The bacteria were cultivated in nutrient agar broth, incubated, and stirred vigorously for three days on a shaker at 30 °C, allowing *P. aeruginosa* to flourish completely. Following incubation, the mixture was centrifuged at 6,000 rpm for 20 min, the bacterial residues were discarded, and the top aqueous layer was transferred to another flask, which can be utilized for IONP biosynthesis.

### Biosynthesis of ionps (Fe_3_O_4_ and Fe_2_O_3_ NPs)

**Fig. 1:**
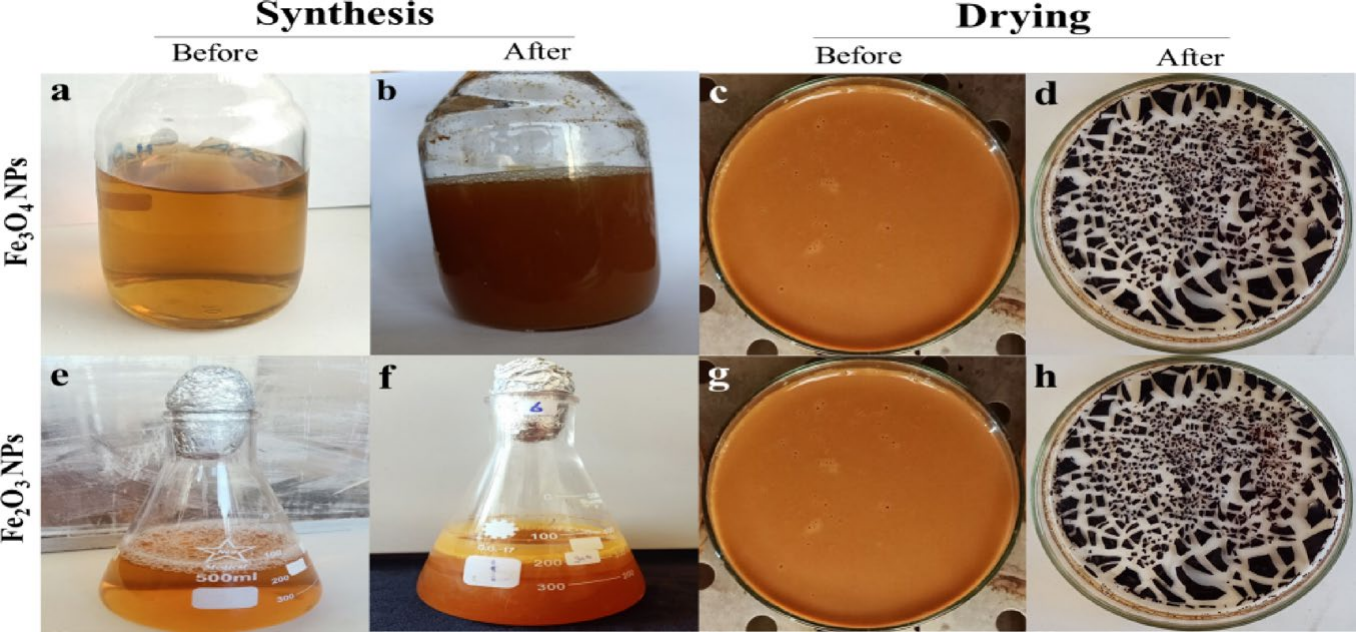
Biosynthesis of IONPs **(a)** before synthesis and **(b)** after synthesis of Fe_3_O_4_ NPs but **(e)** before synthesis and **(f)** after synthesis of Fe_2_O_3_ NPs using *Pseudomonas aeruginosa* kb1 bacterial supernatant. Nano-iron pellets were collected in Petri dishes after centrifugation, **(c)** before and **(d)** after drying in an oven at 80 °C for 20 h for the Fe_3_O_4_ NPs, whereas they were collected **(g)** before and **(h)** after drying for the Fe_2_O_3_ NPs.

For the synthesis of the IONPs, in a 100 ml Erlenmeyer flask with continuous stirring, 50 µl (µl) of aqueous solutions of FeSO_4_.7H_2_O and Fe_2_(SO_4_)_3_.5H_2_O (1 mM) were added to 50 ml of bacterial supernatant with a pH of 7.5 as shown in Fig. 1. The solutions were gently agitated on a shaker for two days at 37 °C. Following the incubation period, the pellets recovered from centrifugation at 10,000 rpm for 10 min were rinsed with deionized distilled water and resuspended to eliminate any contaminants. The pellets were then oven-dried at 80 °C for 20 h before being characterized for further study.

### Preparation of silver-doped ionps (Ag-doped Fe_3_O_4_ and Ag-doped Fe_2_O_3_ NPs)

**Fig. 2:**
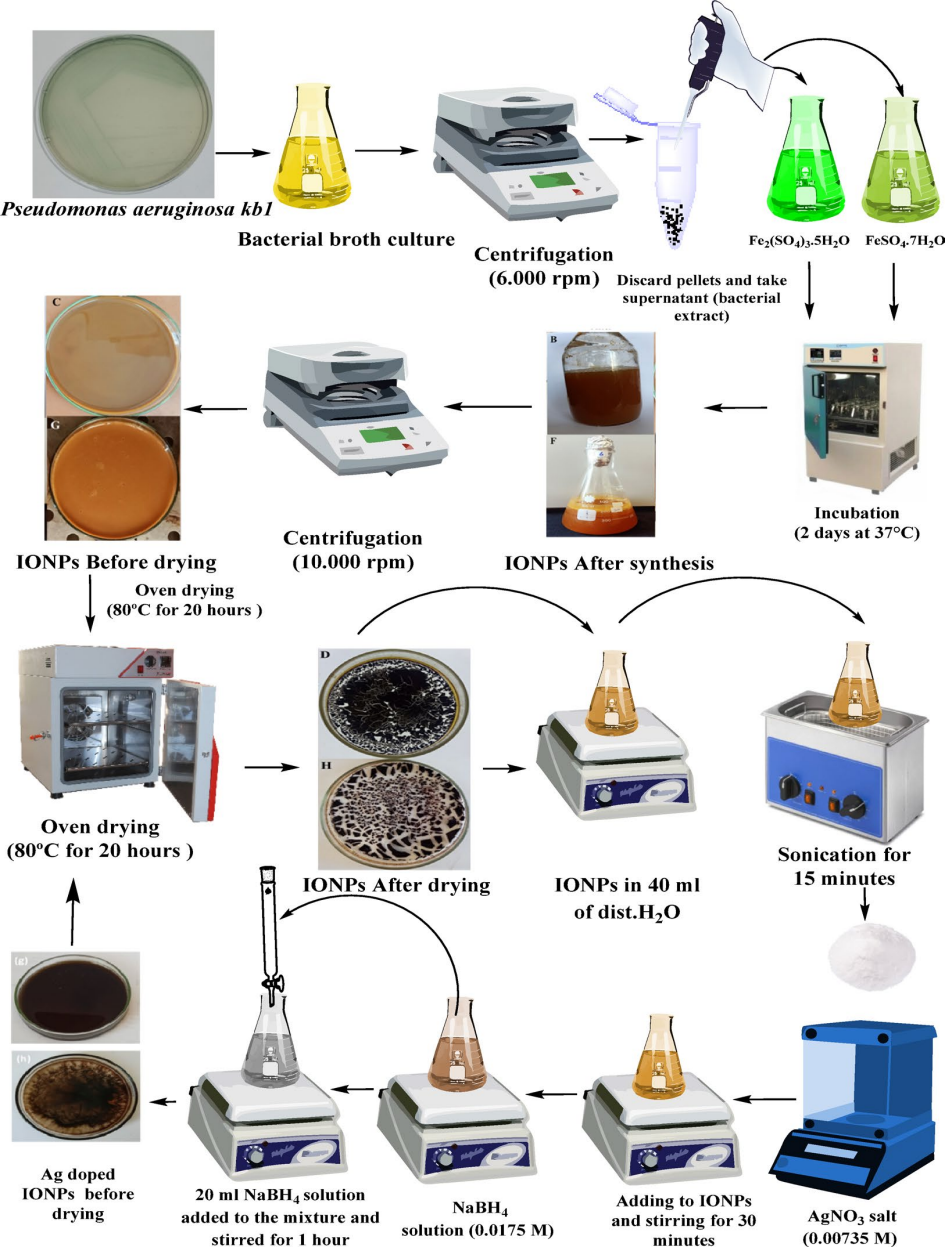
The scheme of synthesis process for IONPs and Ag-doped IONPs.

For the Ag-doped IONP synthesis, chemical reduction is the chosen method for preparing Ag-doped IONPs as reported in previous work by Atacan, Özacar^32^. To the two separated conical flasks 40 mL of distilled water, 0.1 g of Fe_3_O_4_ and Fe_2_O_3_ NPs were sonicated for 15 min, respectively. Next, 0.05 g of Ag NO_3_ powder salt (0.00735 M, calculated as 5% of the MO weight amount is Ag) was added, and the mixture was stirred for 30 min as illustrated in Fig. 2. Within that period, a sodium borohydride NaBH_4_ solution (0.0175 M) was made by stirring NaBH_4_ in 100 ml of frigid, distilled water. After that, 20 ml of NaBH_4_ solution was added to the mixture and agitated at room temperature for 1 h for the reduction of Ag^+^ to Ag^0^ on the surface of the Fe_3_O_4_ and Fe_2_O_3_ NPs. The produced Ag-doped Fe_3_O_4_ and Ag-doped Fe_2_O_3_ NPs were dried in an oven for 20 h at 80 °C.

### Characterization of ionps and silver-doped ionps

The synthesized IONPs and Ag-doped IONPs were investigated via ultraviolet-visible (UV–Vis) spectroscopy, in which the wavelength of scanning ranged from 200 to 800 nm at room temperature. A Jenway UV–Vis spectrophotometer (7205, United Kingdom) was used to determine the maximum wavelength (λ_max_), which confirmed the production either of IONPs, and silver-doped IONPs. Fourier-transform infrared spectroscopy (FTIR) transmittance was studied in the 400–4000 cm^−1^ range using a Burker Vertex 80 (Germany). X-ray diffraction (XRD) studies were employed to evaluate the crystallinity and phase multiplicity of IONPs and Ag-doped IONPs with a scanning range of 10–80°. Combined scanning electron microscopy (SEM) was applied to capture images at high magnification and resolution, revealing the morphology and surface properties of the synthesized IONPs and Ag-doped IONPs. Energy-dispersive X-ray (EDX) spectroscopy was also performed via SEM to characterize the elemental composition of the synthesized NPs. A transmission electron microscope (TEM), (JEOLJEM-2100, USA), was used to assess the size, shape, and homogeneity of the NPs. The zeta potential (Malvern Zeta size Nanozs90, USA) was conducted to evaluate the surface charge of the NPs and the stability of the nanocolloidal solutions. Furthermore, the particle sizes of the IONPs and Ag-doped IONPs were studied via the dynamic light scattering (DLS) technique.

### Biological evaluation of the synthesized ionps and silver-doped ionps

#### Antibacterial assays

The antibacterial activities of the synthesized IONPs and Ag-doped IONPs were investigated against different pathogenic strains. The Gram-positive bacterial strains used included methicillin-resistant *Staphylococcus aureus* (EMCC number 1353 t) and *Staphylococcus aureus* (ATCC 6538), while the Gram-negative bacterial strains used were *Escherichia coli* (ATCC 10536), *Klebsiella pneumoniae* (ATCC 10031), and *Salmonella typhi* (ATCC 25566). Streptomycin was manipulated as a positive control. Each antibacterial method was performed in triplicate and the zone of inhibition was measured and represented as mean ± standard deviation (mean ± S.D).

#### Agar well diffusion method

The agar well diffusion method was used to evaluate the efficacy of the synthesized IONPs and silver-doped IONPs. The zone of inhibition was measured in millimeters (mm) classifying the synthesized NPs as either strong, medium, or weak^33^. A four-well plate was inoculated with 50 µl of sterilized standard inoculum of the bacterial species (OD_600_ of ~ 0.7). Each well was loaded with various concentrations of 2.5, 5, 10, 20, 30, 40, and 50 IONPs or Ag-doped IONPs separately and then incubated for 24 h at 37 °C. After the incubation period, the diameters of the growth inhibition zones around the wells were measured.

#### Growth curves of *E. coli* and *K. pneumoniae*

Growth curves were obtained via a previously described method by Bandyopadhyay, Peralta-Videa^34^ with slight modifications. One hundred µl of freshly activated bacterial culture (*K. pneumoniae* and *E. coli*) was grown in 10 ml of media containing 50, 100, and 200 mg of IONPs, whereas Ag-doped IONPs were 5, 10, 20 mg at 37 °C for 120 h with constant shaking at 150 rpm. Growth was tracked by taking samples after zero, 2, 4, 6, 8, 24, 48, 72, 96, and 120-hour time intervals and then inoculated on nutrient agar plates. The colony-forming units (CFUs) of each bacterial strain were calculated, and the findings obtained are presented as the mean log CFU/ml over various time intervals. Flasks bearing nutritious media and inocula without nanoparticles were employed as reference samples.

#### SEM analysis of *E. coli* and *K. pneumoniae*

To gain direct evidence of the antibacterial behavior of IONPs, SEM micrographs were obtained from *E. coli* and *K. pneumoniae* samples. One hundred microlitres of bacterial suspensions of the *K. pneumoniae* strain (OD_600_ of ~ 1) were inoculated in 10 ml of nutrient broth media supplemented with 50 mg of Fe_3_O_4_ NPs or 100 mg of Fe_2_O_3_ NPs. For *E. coli*, 100 µl of bacterial culture was inoculated in 10 ml of nutrient broth medium with 50 mg of Fe_3_O_4_ NPs or Fe_2_O_3_ NPs. The control samples for each strain contained 10 ml of nutrient broth medium and 100 µl of inoculum without nanoparticles. All the tubes were placed in a shaker at 37 °C for 24 h. In the next step, the four samples and two controls were centrifuged at 6000 rpm for 5 min and washed with sterilized saline solution. This step was repeated three times. The supernatant was subsequently discarded, and the pellets were added to the fixative for SEM analysis.

#### Comet assay of *E. coli* and *K. pneumoniae*

After being inoculated with sub-MIC quantities of the synthesized NPs, the dynamic growth curve data were obtained. *E. coli* and *K. pneumoniae* were cultured with shaking at 37 °C until the mid-log phase of development (OD_600_ of ~ 0.6–0.7). Then, the cells were harvested by centrifugation (5,000 rpm for 5 min), washed twice with saline solution (1 M), and resuspended in 1X phosphate-buffered saline (PBS). For the comet assay protocol, Dhawan et al.. followed with slight modifications^35–37^. Briefly, 2 µl of bacterial cells before and after treatment with IONPs (Fe_3_O_4_ NPs and Fe_2_O_3_ NPs) and Ag-doped IONPs (Ag-doped Fe_3_O_4_ NPs and Ag-doped Fe_2_O_3_ NPs) were mixed with 200 µl of 0.75% agarose prepared in 0.1X PBS. A microscope slide previously coated with a thin film of 0.75% agarose was loaded with 100 µl of bacterial cells soaked in agarose solution and solidified by incubation for 30 min at 4 °C. Afterwards, the slides were flooded with 200 µl of third-layer agarose solution and reincubated at 4 °C until they solidified. The slides were then incubated at 37 °C for one hour to allow for cell lysis. The cells were then subjected to enzyme digestion for two hours at 37 °C. After digestion of the cell wall with lysis and enzyme treatment, the slides were subjected to electrophoresis for 50 min at 12 V and 300 mA. Following electrophoresis, the slides were subjected to staining with a newly prepared ethidium bromide solution. The comets were examined with a fluorescence microscope (Olympus BX 43 F) connected to a CCD camera. The tail length was detected and analyzed via the CaspLab program.

#### Anticancer and cytotoxicity assays

The anticancer and cell cytotoxicity for the human cell lines were investigated using MTT assay. This was evaluated by the reduction of yellow MTT to purple formazan in the presence of mitochondria was used to test cell viability^38^. Colon cancer cells (HCTT166), lung cancer cells (A549), and a retina cell line (RPE1) from ATCC USA were incubated in DMEM-F12 media supplemented with 1% L-glutamine and 1% antibiotic-antimycotic combination at 5% CO_2_ and 37 °C for 10 days before being seeded in new growth media on microtiter plastic plates with 96-well plates at a concentration of 10 × 10^3^ cells/well for 24 h at 37 °C with 5% CO_2_. The nutrient mixture was removed, fresh medium was added, and the cells were allowed to grow either as a negative control or with different concentrations of the synthesized NPs with overall concentrations of 100-50-25-12.5-6.25-3.125-0.78–1.56 µg/ml. After 48 h of incubation, the medium was removed. 40 µl of MTT salt (2.5 µg/ml) was injected into each well and incubated for an additional four hours at 37 °C. To terminate the reaction, 200 µL of 10% SDS was pumped into each well and left overnight at 37 °C. A positive control (doxorubicin) at 100 µg/ml was utilized as a recognized natural cytotoxic substance^39,40^. The absorbance was then determined at 595 nm and 620 nm via a microplate multiwell reader. An independent t-test was conducted via the SPSS 11 program to determine the statistical significance between samples and negative controls. The vehicle utilized to dissolve the synthesized NPs was DMSO, which had a final concentration of less than 0.2% in the cells.

## Results and discussion

### Characterization of the synthesized ionps and silver-doped ionps

#### UV–Vis absorption spectra analysis

**Fig. 3:**
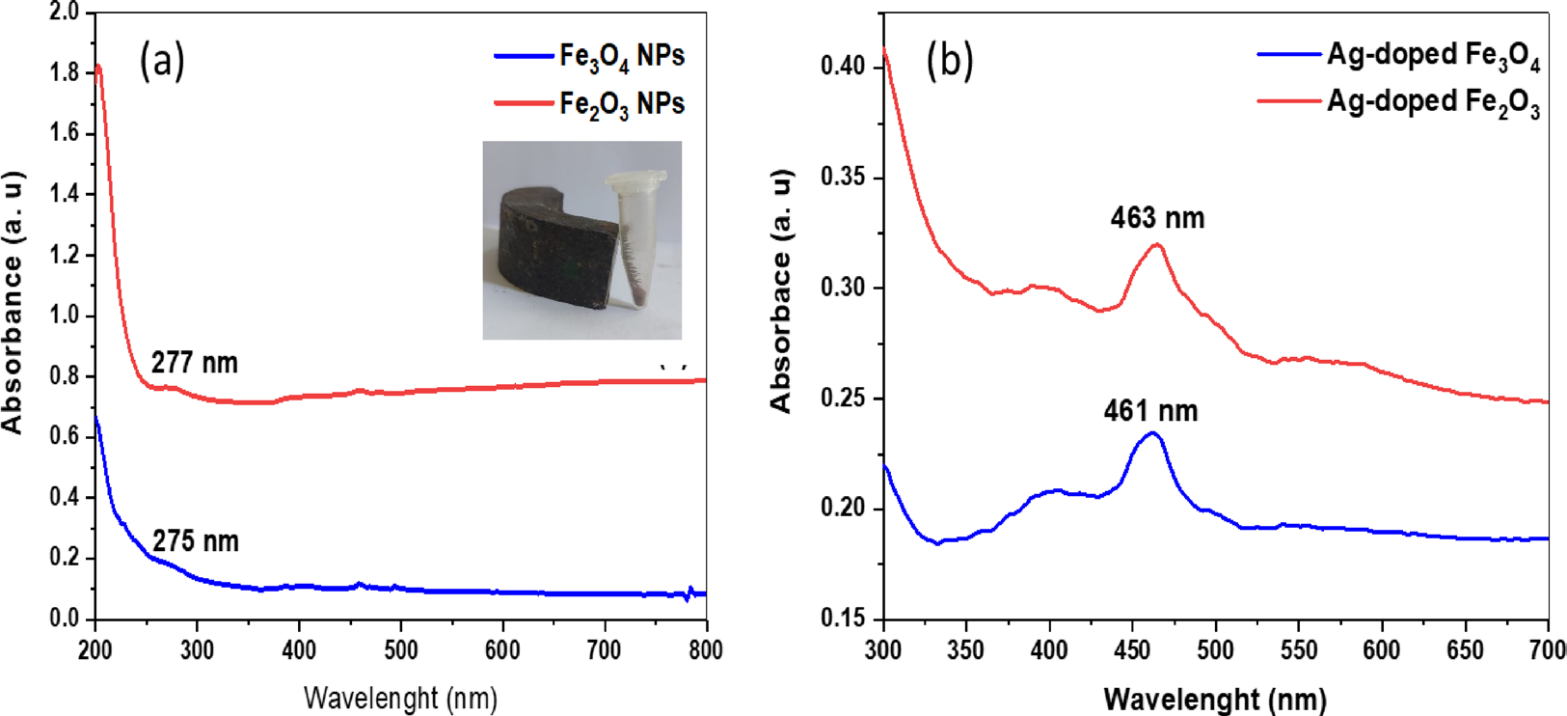
UV–visible absorption spectra of **(a)** Fe_3_O_4_ NPs and Fe_2_O_3_ NPs synthesized from *P. aeruginosa kb1*, inset the photograph of the Fe_3_O_4_ NPs attracted by a magnet, and **(b)** Ag-doped Fe_3_O_4_ NPs and Ag-doped Fe_2_O_3_ NPs.

The change in color to dark brown after the synthesis of IONPs is the first optical indication of the success of the synthesis process **(**Fig. 1**).** Recent studies using plant extracts have shown a similar type of color change in FeSO_4_ salt solution during the synthesis of IONPs^41,42^. The UV absorption spectrum of the synthesized Fe_3_O_4_ NPs revealed a small characteristic band at 277 nm, whereas the spectrum of the biosynthesized Fe_2_O_3_ NPs revealed a surface plasmon resonance (SPR) band at approximately 275 nm (Fig. 3a**).** Nanoparticles have great significance in many areas of biomedicine, electronics, optics, and technology. They essentially serve as a link between atomic or molecular structures and bulk materials. Even though they are derived from the same element, metal nanoparticles (MNPs) are distinct from bulk metals. The optical, chemical, and electrical characteristics of NPs are size-dependent. However, by altering the synthesis conditions, these characteristics can be altered. These special characteristics are usually found in metals whose valence orbitals have loosely bonded electrons. This is the outcome of SPR, which is advantageous to nanotechnology. The unique characteristics of MNPs are thus mostly due to the SPR effect^43^. The photograph of the Fe_3_O_4_ NPs attracted by a magnet is depicted (inset image) in Fig. 3a which confirms the magnetic effect of the Fe_3_O_4_ NPs mediated by the bacterial extract as reported by Yadav et al., (2022)^44^.

These peaks are related to Fe residues and the collective oscillation of the Fe surface plasmons. These SPR absorption bands are found in iron nanoparticles^45^. Although, the exact mechanism for the synthesis of IONPs by bacteria has yet to be fully understood. However, reports suggest that nanoparticles are usually formed via a reduction process: ions of metal are initially captured on the surface or within bacterial cells. This material is subsequently reduced to nanoparticles in the presence of biological enzymes^46^. The NaBH_4_ solution served as the reducing agent for the synthesis of Ag-doped IONPs using the chemical reduction process. Upon the addition of NaBH_4_ solution to the mixture of IONPs and AgNO_3_, the color immediately changed to a greyish-brown hue. This is the first sign that Ag-doped iron NPs are being made. Also, **Mangala Nagasundari**,** Muthu**^47^ demonstrated a comparable shift in color as represented in Fig. 2.

For the Ag-doped Fe_3_O_4_ NPs and Ag-doped Fe_2_O_3_ NPs, a broad absorption band can be observed within 380–480 nm, with maximum absorption peaks at 461 and 463 nm, respectively. These findings are consistent with earlier studies^48–51^, which showed that the peak attributed to Ag NPs is extensively reported to be between 390 and 580 nm. Silver has a high efficiency of light absorption and scattering because of a strong interaction known as surface plasmon resonance, which is caused by the conduction electrons on the silver surface oscillating collectively when excited by a particular light wavelength^52^ (Fig. 3b).

Research on the large-scale synthesis of doped NPs for biological applications is rapidly increasing. Owing to their photooxidizing and photocatalytic features, doped NPs are employed primarily as antibacterial agents in biomedical applications. **Abdulkadhim**^53^ studied the antibacterial activity of pure and silver-doped TiO_2_ NPs produced via a hydrothermal technique against *E. coli* and *S. aureus*. It became apparent that Ag-doped TiO_2_ NPs were more effective as antibacterial agents. Anti-parasitic and anticancer medications also use doping. Several doping substrates are being employed because of their antitumor properties. The Ag^+^/Fe^2+^ co-doped with nanohydroxyapatite nanocomposites allowed for the administration of medicine at many stages in specific areas, which could be used for treating malignant tumours with no bacterial side effects^54^. Theranostic applications have recently made use of the concept of doping. The delivery of drugs is one of the most common uses of doping. It has undergone many modifications to make it appropriate for medication delivery. After being synthesized and tested as theranostic cancer probes, 131 iodine-doped Ag-PEG NPs were found to be safe for normal cells below a certain dosage, suggesting that they may be utilized for both diagnosis and treatment^55^. Several monodispersed nickel-doped IONPs with superparamagnetic properties were created by **Lu**,** Xu**^56^; these NPs allowed for high-resolution T_1_-weighted and T_2_-weighted dual-mode MR imaging in vivo with an extended circulation duration. High colloidal stability and enhanced biosafety were observed, indicating the possible use of nickel-doped iron oxide nanoparticles for accurate diagnosis in deep-tissue T_1_-T_2_ dual-mode MR imaging.

#### FT-IR spectroscopy analysis

**Fig. 4:**
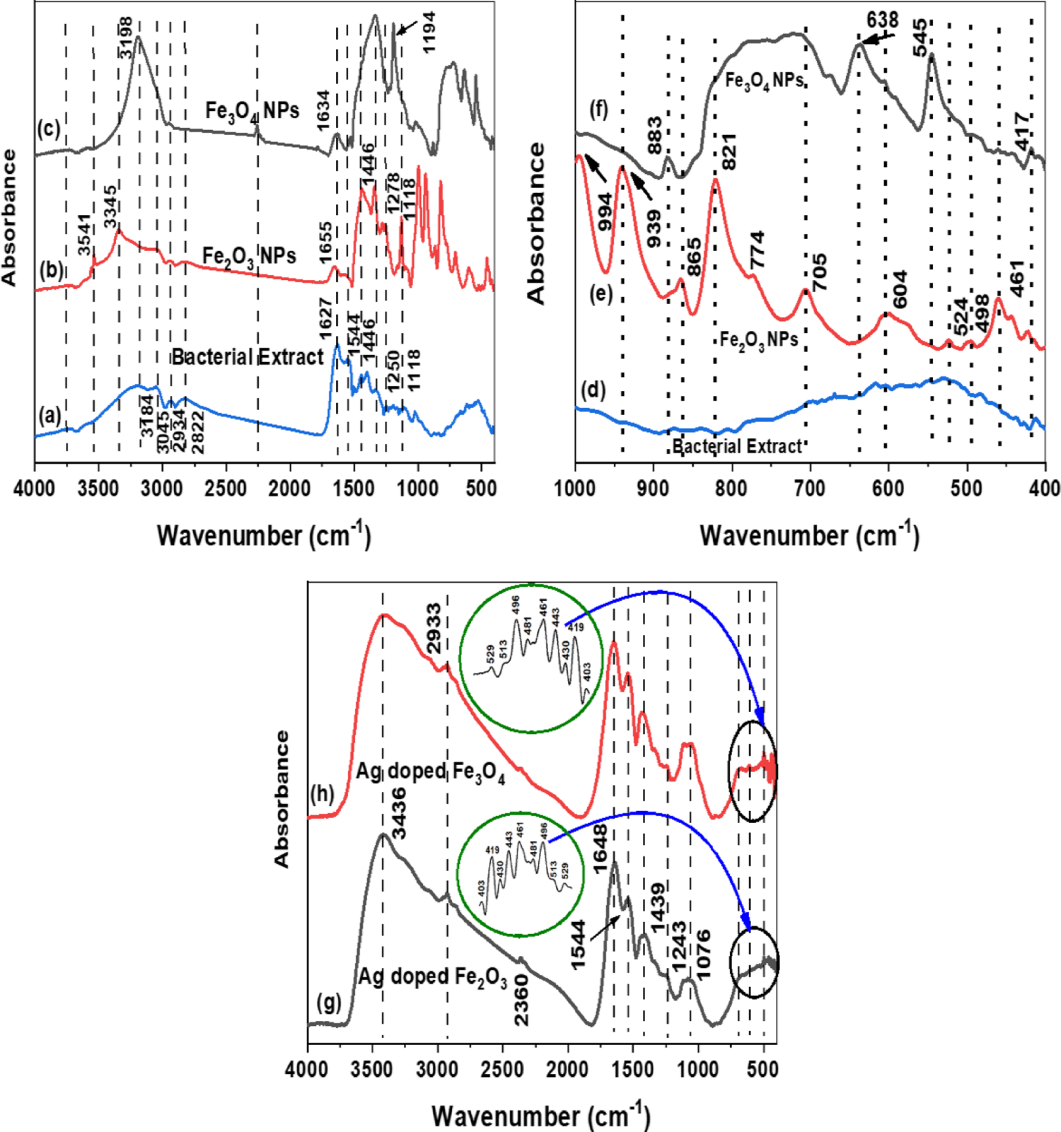
FTIR spectra of the IONPs synthesized in green via magnetotactic bacteria and silver-doped IONPs: **(a)** bacterial extract, **(b)** Fe_2_O_3_ NPs, **(c)** Fe_3_O_4_ NPs, **(d)** bacterial extract with a wavenumber range of 400–1000 cm^−1,^ **(e)** Fe_2_O_3_ NPs with a wavenumber range of 400–1000 cm^−1,^ **(f)** Fe_3_O_4_ NPs with a wavenumber range of 400–1000 cm^−1,^ **(g)** Ag-doped Fe_3_O_4_ NPs, and **(h)** Ag-doped Fe_2_O_3_ NPs.

The results of the FT-IR analysis of the *P. aeruginosa* bacterial extract are illustrated in Fig. 4a. The spectral range between 3800 and 2800 cm^−1^ has many bands. One of these bands is located at 3757 cm^−1^, which corresponds to the stretching mode of the OH ^–^ group, suggesting the presence of both bonded and non-bonded hydroxyl groups. The presence of asymmetrical and symmetrical C-H stretching, indicating an aliphatic methylene group, was observed at 2934 cm^−1^ and 2822 cm^−1^. The presence of amino, amide, carboxyl, phospholipid, carbonate, and sulfate groups was detected based on the occurrence of certain peaks at their corresponding positions. The interaction of IONPs with hydroxyl, carboxyl, and amino groups was confirmed by the different shifts in the peak locations detected in the spectra of *P. aeruginosa*, as shown in Fig. 4a. Moreover, surfactants, including both anionic and non-ionic surfactants, induce changes in the composition of the cell wall and cytoplasmic membrane. These changes involve alterations in the amounts of carbohydrates, lipids, proteins, and nucleic acids present in the cell wall and cytoplasm. Furthermore, three characteristic bands at 1118, 1446, and 1544 cm^−1^ due to the C − O stretching of the ester groups, the stretching vibration of COO − of carboxylic acid groups, and the N‒H bending of the amino groups of the amino acid of the bacterial extract were assigned. However, surfactants do not affect the structural interactions, such as hydrogen bonding, between these biomolecules. The hydroxyl, carbonyl, carboxyl, sulfonate, amide, imidazole, phosphonate, and phosphodiester groups were identified as the primary functional groups involved in the biosorption process^57,58^. Several of these groups were observed on the outer membrane of *P. aeruginosa* cells and have the potential to interact with the metal oxide NPs, as depicted in Fig. 4b **and c**. Furthermore, many cross-peaks were observed between 650 and 1750 cm^−1^, which can be attributed to changes in carbohydrates, lipids, and proteins. Both the synchronous and asynchronous spectra exhibit significant cross peaks associated with -OH groups.

The FTIR spectra of the bacterial extract (a), Fe₂O₃ NPs (b), and Fe₃O₄ NPs (c) all have multiple overlapping peaks. The most noticeable ones are at 3445–3541 cm⁻¹ (O–H/N–H stretching), 1634–1655 cm⁻¹ (C = O stretching), and 1118–1194 cm⁻¹ (C–O stretching). These peaks can be considered in both the pure extract and the nanoparticle samples. This clearly shows that organic molecules from the extract are adsorbed onto the surfaces of the nanoparticles, perhaps acting as capping and stabilizing agents.

The vibrational frequency of the C = O group shifted from 1627 cm⁻¹ to 1655 cm⁻¹ for Fe₂O₃ NPs and from 1627 cm⁻¹ to 1634 cm⁻¹ for Fe_3_O_4_ NPs. We would like to clarify that the presence of these peaks in the nanoparticle spectra indicate the reducing strength of the extract^59,60^. It also shows how the physical and chemical properties of the extract components interact with the nanoparticles once they are made. This is something that happens a lot in green synthesis methods, where the biological extract does a lot of different things, like reducing, capping, and stabilizing^59^.

Figures 4d, e, and f show the FTIR spectra of both the Fe_2_O_3_ NPs and the Fe_3_O_4_ NPs compared with those of the bacterial extract. The Fe–O bond is responsible for the characteristic absorption peaks at 461 cm^−1^, 524 cm^−1^, 605 cm^−1^, 705 cm^−1^, 821 cm^−1^, 865 cm^−1^, 939 cm^−1^, and 994 cm^−1^^61,62^. The bands between 400 and 650 cm^−1^ correspond to Fe–O bonds in the stretching and vibration modes. The Fe_3_O_4_ NP spectrum shows bands at 417 cm^−1^, 545 cm^−1^, 638 cm^−1,^ and 883 cm^−1^, indicating that the Fe‒O bonds of the magnetite nanoparticles are comparable to the obtained findings by **Basavegowda**,** Mishra**^63^. The metal-oxygen band at 517 cm^−1^ is attributed to metal intrinsic stretching vibrations at the tetrahedral site, whereas the band at 461 cm^−1^ represents Fe‒O octahedral-metal stretching^64–66^.

The FTIR spectra of the Ag-doped Fe_2_O_3_ and Ag-doped Fe_3_O_4_ nanocomposites are shown in Fig. 4 g and h. The presence of different vibrational absorption peaks between 400 and 700 cm^−1^ for both synthesized doped NPs is depicted in the inset of Fig. 4g and h. These peaks correspond to the metal-oxygen absorption band stretching vibration (Ag‒O and Fe‒O bonds), indicating the existence of maghemite (γ‒Fe_2_O_3_) and magnetite nanoparticles alongside silver and/or silver oxide nanoparticles. Furthermore, interactions among IONPs and nanocomposites can alter their intensities. The hydroxyl group peaks can be attributed to the bending vibration of the OH band absorption of water molecules on the surface of the IONPs in the presence of the Ag-doped IONPs; C–H (asymmetric and stretching), C = O stretching vibration, COO − symmetric stretching, and C–C stretching vibrations have been displaced to 3429, 2940, 1641, 1536, 1417, and 1062 cm^−1^, respectively, in comparison with the bacterial extract spectrum^67–71^. Based on the FTIR results, ferric oxide (γ-Fe_2_O_3_) and magnetite (Fe_3_O_4_) NPs were synthesized with the aid of cell-free extract filtrate biological components. This is in addition to the Ag-doped IONPs. More discussion will be conducted in the next section via XRD analysis and electron spectroscopy imaging techniques.

**Fig. 5:**
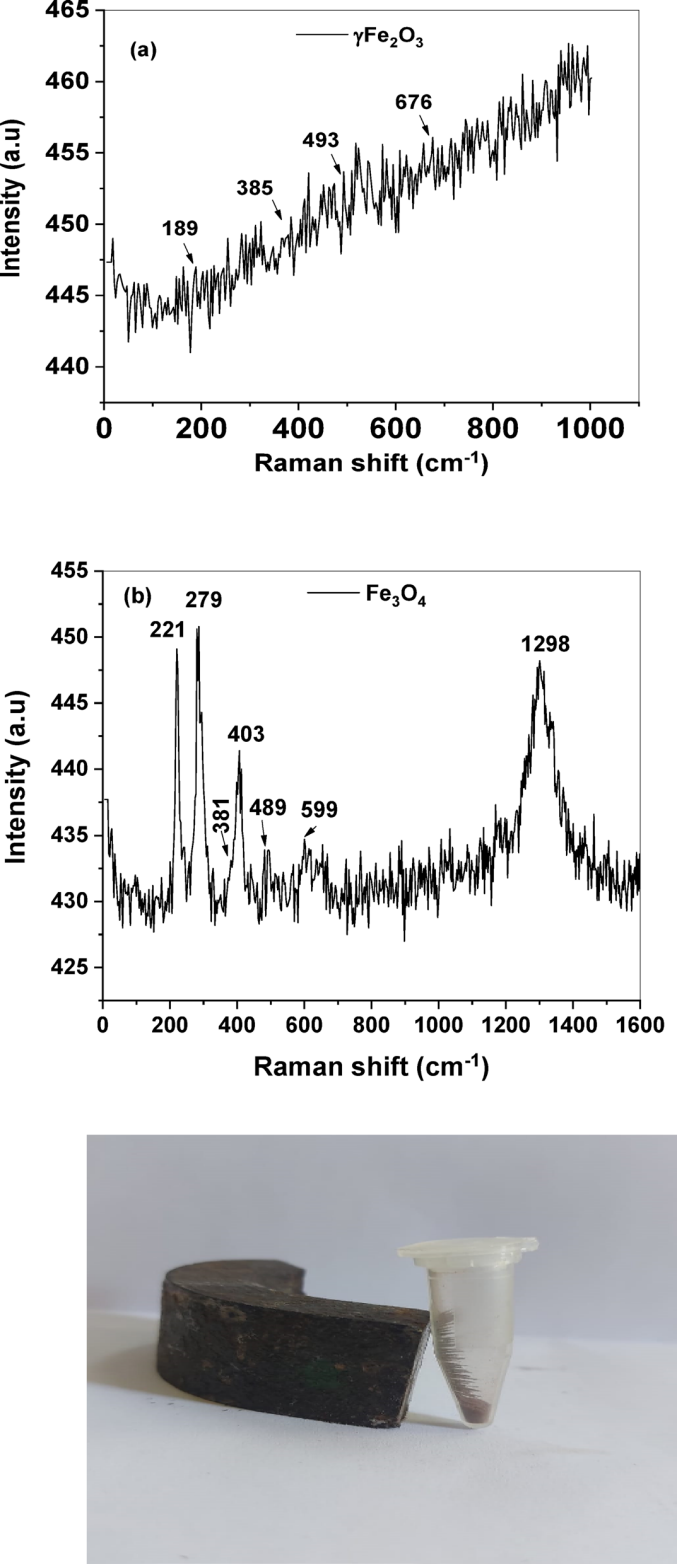
FTIR spectra of the IONPs synthesized in green via magnetotactic bacteria: **(a)** Fe_2_O_3_ NPs, and **(b)** Fe_3_O_4_ NPs, and **(c)** digital photograph of Fe_3_O_4_ NPs powder attraction by a magnet.

From the Raman shift spectrum of Fe_2_O_3_ NPs, several distinct wavenumber shifts were assigned 189, 385, 493, and 676 cm^−1^ as shown in Fig. 5a. These findings were inconsistent with the results obtained by Hai et al. (2008)^72^. On the other hand, the Raman shift wavenumbers for the Fe_3_O_4_ NPs mediated by the bacterial extract (Fig. 5b) were 221, 279, 381, 403, 489, 599, and 1298 cm^−1^, respectively as reported by Yew et al. (2017)^73^. The photograph of the Fe_3_O_4_ NPs attracted by a magnet is depicted in Fig. 5c which confirms the magnetic effect of the Fe_3_O_4_ NPs mediated by the bacterial extract as reported by Yadav et al., (2022)^44^.

#### XRD analysis

**Fig. 6:**
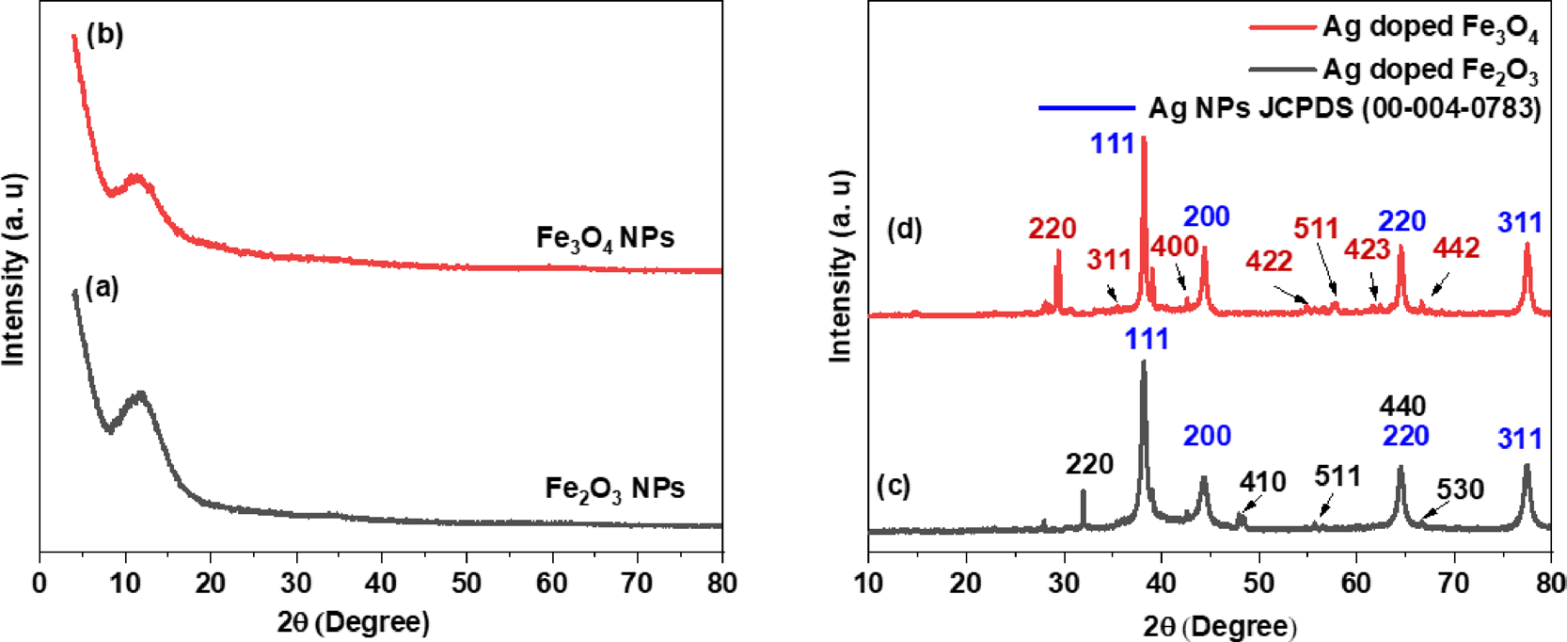
XRD patterns of the synthesized NPs **(a)** Fe_2_O_3_ NPs, **(b)** Fe_3_O_4_ NPs, **(c)** Ag-doped Fe_2_O_3_ NPs, and **(d)** Ag-doped Fe_3_O_4_ NPs.

Additional investigation of the phase and crystalline structure of the produced NPs was conducted using XRD, Fig. 6. The pattern indicated the amorphous nature of the green synthesized NPs in both the Fe_2_O_3_ and Fe_3_O_4_ NP samples (Fig. 6a and b), which was also in accordance with the literature^74–76^ and indicated their amorphous structure. However, insufficiently clear diffraction peaks were observed in the pattern. The broad peak that appears at 2θ values of 10° to 20° may be due to the organic components coated from the reaction media that are in charge of stabilizing the synthesized nanoparticles^74–76^.

The XRD spectrum of the synthesized Ag-doped Fe_2_O_3_ NPs is shown in Fig. 6c. An XRD study of the desired NPs revealed that the synthesized Ag-doped Fe_2_O_3_ NPs were crystalline and were in good accordance with the JCPDS database number (00–039–1346)^77^. The diffraction angles (2θ) and corresponding planes were observed at 31.96° (220), 48.11° (410), 64.55° (440), and 66.72° (530), which were attributed to the maghemite (γ-Fe_2_O_3_) cubic crystalline structure. In addition, for the Ag-doped Fe_2_O_3_ NP X-ray pattern, many other diffraction peaks equal to 38.20°, 44.31°, 64.67°, and 77.44° can be well matched with the cubic phase structure and correspond to the (200), (220), and (311) crystal planes, respectively, of the cubic Ag NPs, which are well matched with the JCPDS card number (00-004-0783)^67^. There is an overlap of the peak of the Ag NPs and Fe_2_O_3_ NPs at 64.55°, with a plane of (220) that conforms to the formation of Ag-doped Fe_2_O_3_ NPs. The synthesized Ag-Fe_2_O_3_ was crystalline and was in good accordance with the JCPDS database number reported (01-071-3762)^70,71^. Furthermore, the XRD pattern of the synthesized Ag-Fe_3_O_4_ NPs **(**Fig. 6d**)** shows several diffraction peaks at 2θ = 29.24° (220), 35.63° (311), 42.54° (400), 55.03° (422), 57.75° (511), 62.38° (423) and 66.72° (442), which agree well with the XRD pattern of JCPDS file no. (00-072-2303)^69,78–81^. NaBH_4_ is known to be a potent reducing agent for AgNO_3_ salt, resulting in Ag and/or Ag_2_O NPs. Furthermore, **Bae**,** Gim**^82^ reported that the creation of smaller NPs with NaBH_4_ might be attributable to either the chemical etching process on the layer of iron oxide or the oxidative disintegration of IONPs. A negative change in the redox potential of metallic nanoparticles is widely believed to be caused by the adsorption of borohydride on the surface of the particles. Because of their high vulnerability to oxygen oxidation, metals may undergo oxidative dissolution as a result of this change^83–85^. When the oxygen content subsequently falls below a certain limit, the metal ions in the solution can be reduced by NaBH_4_ to produce novel NPs, as previously proven by silver nanoparticles^83^. The addition of NaBH_4_ resulted in the formation of smaller Ag NPs in an oxygen-free environment, demonstrating that oxidative dissolution plays a non and/or weak role the reduction of the remaining unreacted iron salt traces. Howecer, De Resende et al. (2006)^86^ indicated that the incorporation of sodium borohydride (NaBH_4_) into the iron salt led to the estimation of goethite (α-FeOOH) formation, as evidenced by the XRD pattern observed at a diffraction angle of 2θ = 42.5° for the synthesized iron oxide.

Conversely, this study shows that following the addition of NaBH_4_, the goethite peak (2θ = 42.5°) is absent, indicating an interaction with Boron oxide (B_2_O_3_). Consequently, these findings provide strong evidence that NaBH_4_ plays a limited role in the chemical reduction process for the unreacted iron salt traces that were not reduced by the *P. aeruginosa* bacterial extract.

It seems that the drying duration of 24 h at ~ 100 °C, affects the transformation of the presence of IONPs from the amorphous to crystalline phases^87^. Moreover, the residual of the dissociated NaBH_4_ alters the pH of the mixture of Ag-doped IONPs due to the effluent of OH^−^ groups making it more alkaline. Lin Yu, Michael, and A. Matthews (2011)^88^ reported the effect of temperature on the reaction, with an initial NaBH_4_ concentration of 15 wt%. It is seen that at 25 °C the NaB(OH)_4_ concentration reaches only 1.53 mol/L after 24 h. However, as the temperature increases, the reaction rate and conversion are significantly increased. The reaction rate is the greatest at 80 °C and 100% conversion is attained after about 10 h. As a result, we have evidence that chemical etching processes may occur on the surfaces of magnetite, causing microsized magnetite to break down into nanosized magnetite. The magnetism-induced self-assembly of magnetite facilitates this process even further^89^.

#### SEM analysis

**Fig. 7:**
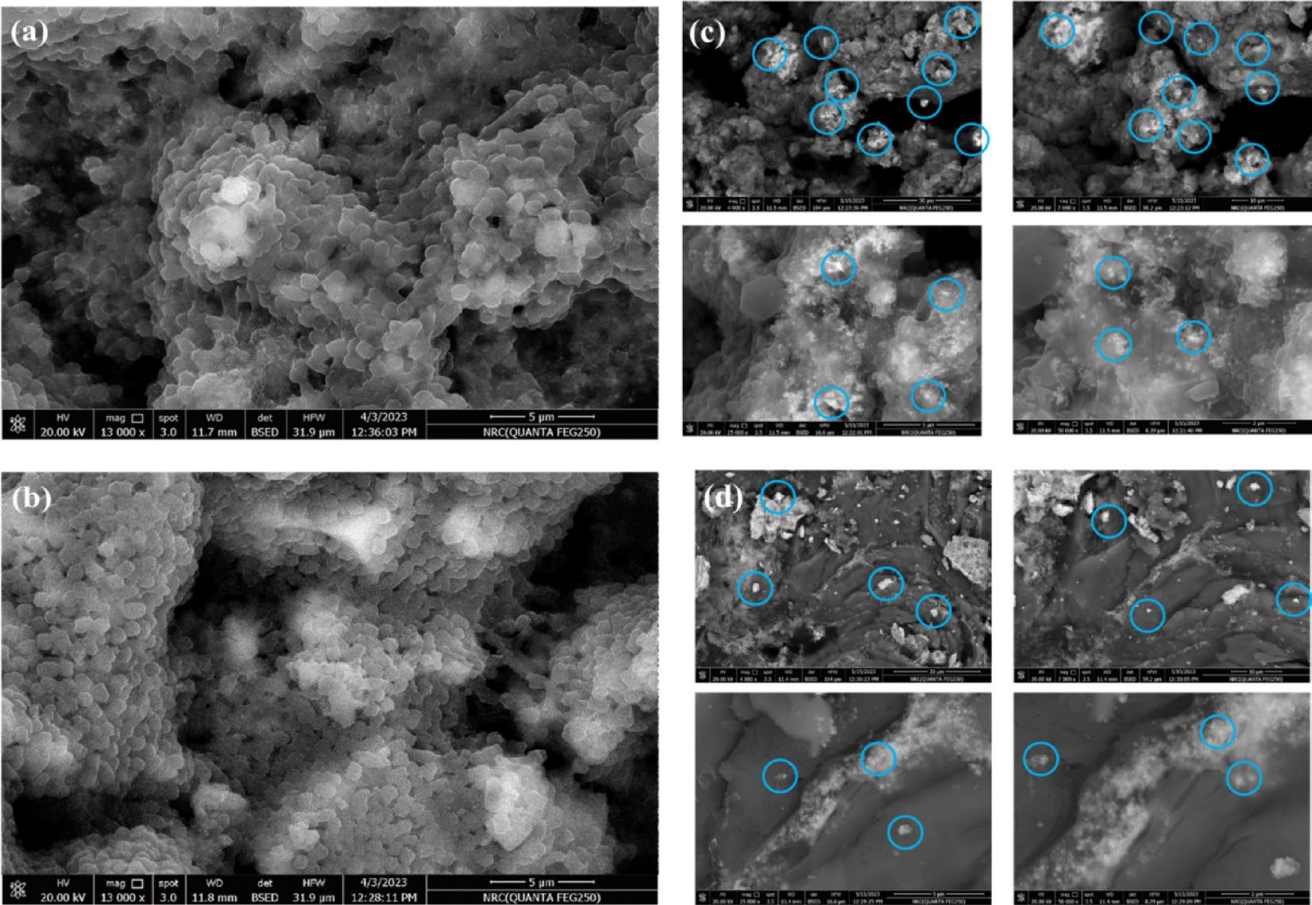
SEM micrographs of the synthesized NPs: **(a)** Fe_3_O_4_ NPs, **(b)** Fe_2_O_3_ NPs, **(c)** Ag-doped Fe_3_O_4_ NPs at different magnifications, **(d)** Ag-doped Fe_2_O_3_NPs.

Figure 7a shows an SEM image of the Fe_3_O_4_ NPs, which reveals that the produced nanoparticles have uneven aggregation and a porous spherical-like structure^90,91^. The morphology of the biosynthesized Fe_2_O_3_ NPs was observed via SEM analysis, as shown in Fig. 7b. Generally, nanocomposites are composed of amorphous, spherical, and agglomerated grains with sizes ranging from 0.6 to 1.1 μm^91,92^. The electrostatic interaction between the surface layers of the nanoparticles may have caused the observed agglomeration of IONPs^80^. SEM micrographs of the Ag-doped Fe_3_O_4_ NPs and Ag-doped Fe_2_O_3_ NPs revealed that the deposited nanoparticles glittered white specks with irregular clusters, rough surfaces, tiny agglomerations, and quasispherical shapes^93^ (Figs. 7c and d).

#### EDX analysis

**Fig. 8:**
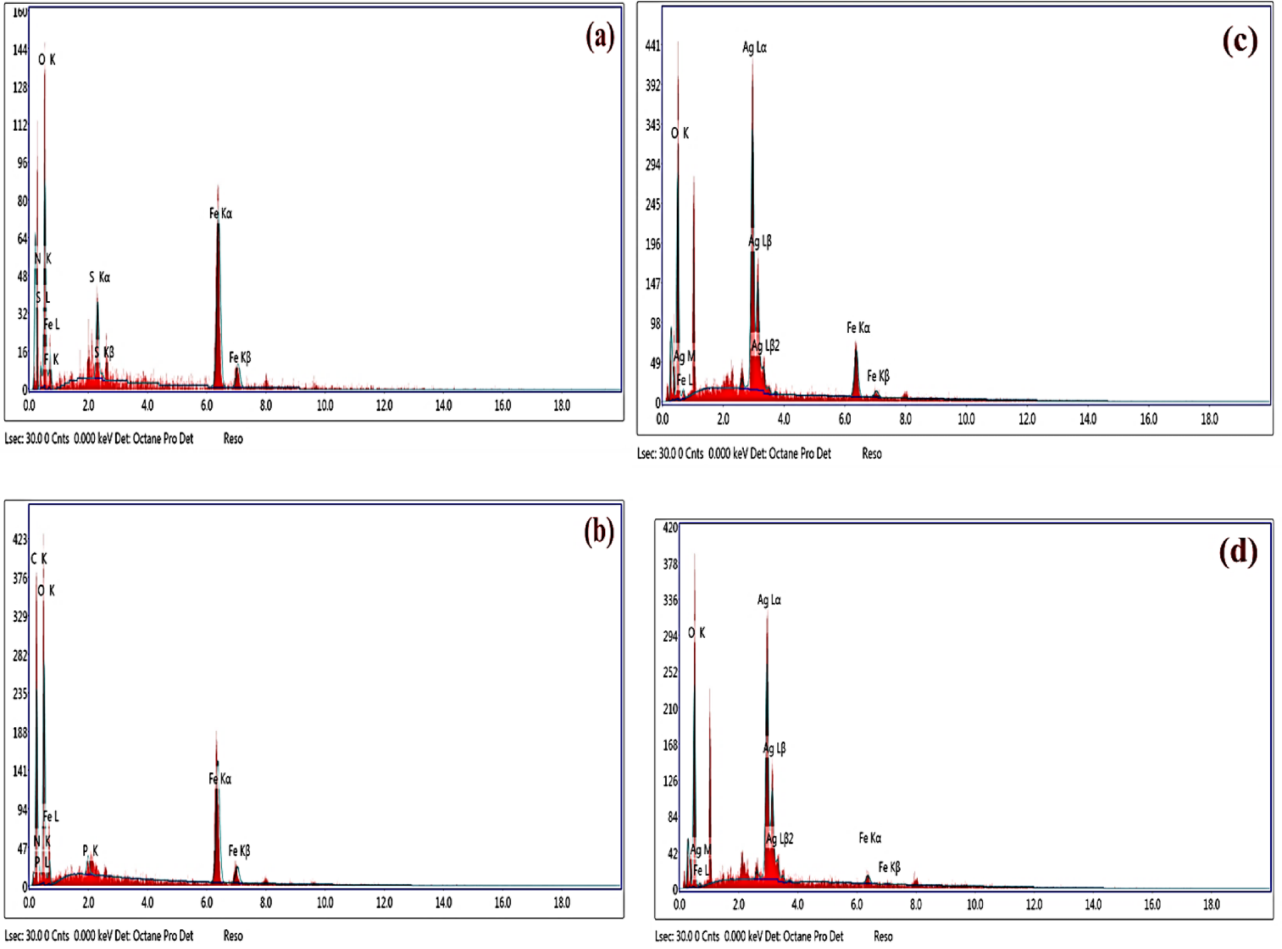
EDX analysis of the synthesized NPs **(a)** Fe_3_O_4_ NPs, **(b)** Fe_2_O_3_ NPs, **(c)** Ag-doped Fe_3_O_4_ NPs and **(d)** Ag-doped Fe_2_O_3_ NPs.

From the EDX spectrum of the Fe_3_O_4_ NPs **(**Fig. 8a**)**, two separate spectral regions captured four distinct strong signals. For oxygen, the region was between 0 and 1 keV, whereas for iron, it was between 0 and 1 keV and between 6 and 7 keV^75^. The peaks represent the binding energies of iron at approximately 0.8, 6.2, and 6.9 keV, whereas the binding energies of oxygen are revealed by the peak at 0.5 keV^66,94^. As a result, the EDX spectrum revealed the existence of Fe_3_O_4_. The iron and oxygen contents were calculated to be 53.26 and 32.57%, respectively. There were minor contaminants of N and S in the samples, which were N and S, from the prepared salts. Sulfur is usually observed during the synthesis of iron oxide NPs from ferric sulfate^95^. The elemental composition of the Fe_2_O_3_ NPs was analyzed via EDX, as shown in Fig. 8b. Iron was found to be present. The EDX spectral peaks at 0.7, 6.4, and 7.0 keV revealed the existence of iron peaks at each of these three locations. The iron, oxygen, carbon, chloride, and phosphorus contents were calculated to be 21.04, 35.39, 33.92, and 0.76%, respectively. C, N, and P are present in the media ingredients on the NP surface. Nevertheless, the O was attributed to the formation of Fe oxides in the Fe_2_O_3_ NPs^96,97^. Similar results were obtained^98^ showing the presence of Fe and O peaks in the EDX analysis.

The EDX spectrum of the synthesized Ag-doped Fe_3_O_4_ NPs is represented in Fig. 8c, with metallic Ag binding energies of approximately 0.6, 2.9, 3.2, and 3.8 keV and Fe binding energies of 0.8, 6.2, and 6.9 keV, respectively. In the Ag-doped Fe_2_O_3_ EDX spectrum **(**Fig. 8d**)**, the silver binding energies are also found at 0.6, 2.9, 3.2, and 3.8 keV, whereas the Fe NP binding energies are represented by peaks at 0.6, 6.4, and 7 keV. The elemental makeup of the bimetallic nanoparticles was evaluated via EDX, which verified that the Ag-doped Fe_3_O_4_ NPs consisted of 36.95% Ag, 12.59% Fe, and 50.46% O, and the Ag-doped Fe_2_O_3_ NPs consisted of 38.53% Ag, 2.95% Fe, and 58.52% O^99,100^.

#### TEM and selected area electron diffraction (SAED) patterns

**Fig. 9:**
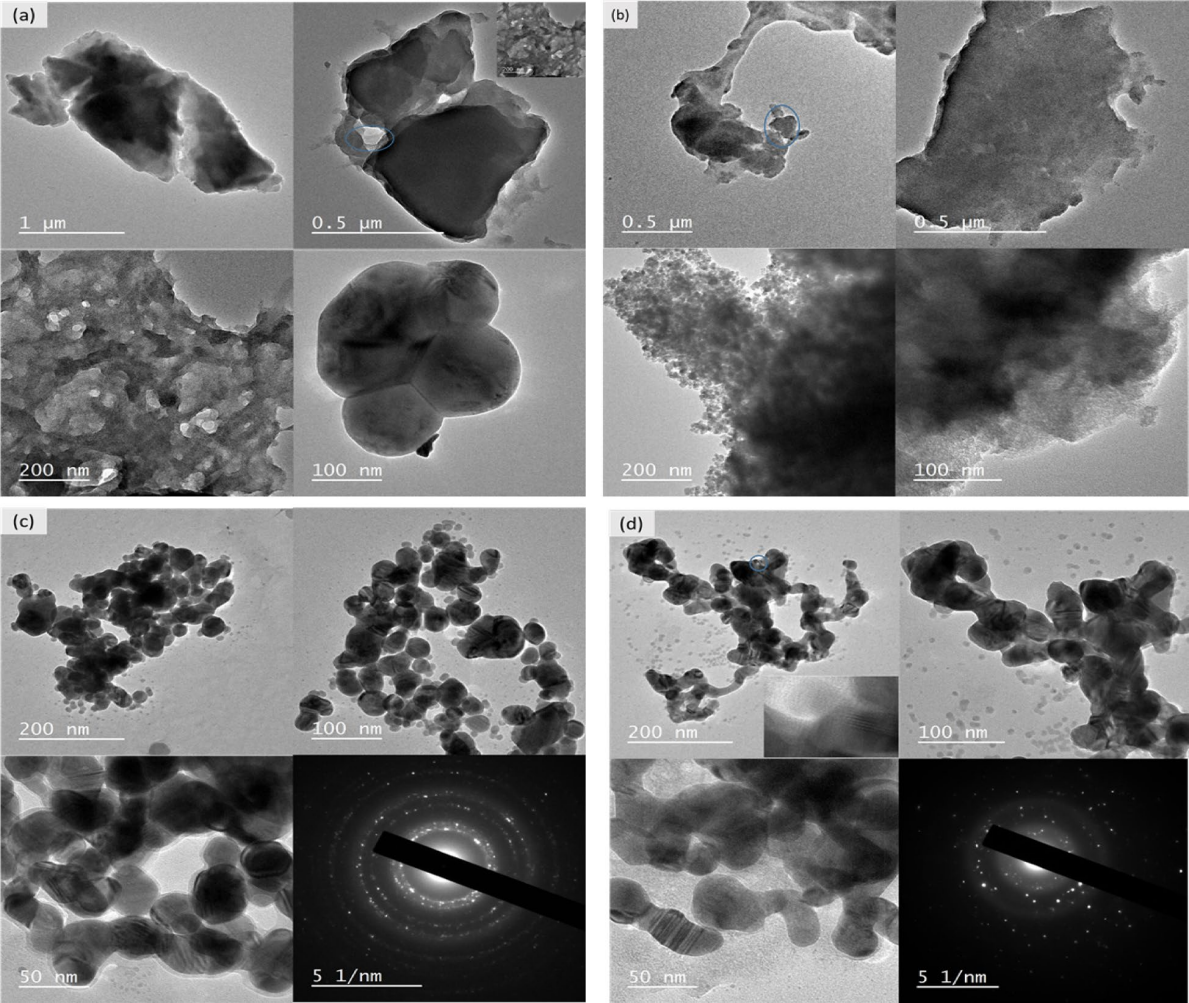
Typical TEM images of the synthesized NPs at various magnifications: (a) Fe_3_O_4_ NPs, 1 μm, 0.5 μm, 200 nm, and 100 nm; (b) Fe_2_O_3_ NPs, 0.5 μm, 200 nm, and 100 nm; (c) Ag-doped Fe_3_O_4_ NPs, 200 nm, 100 nm, 50 nm; and equivalent SAED of Ag-doped Fe_3_O_4_ NPs; (d) Ag-doped Fe_2_O_3_ NPs, 200 nm, 100 nm, 50 nm; and equivalent SAED of Ag-doped Fe_2_O_3_ NPs.

Figure 9a shows micrographs of the Fe_3_O_4_ NPs at magnifications of 1.0 mm, 0.5 mm, 200 nm, and 100 nm, with both spherical and cuboidal-shaped nanoparticles ranging in size from 100 nm to 1000 nm. These findings were verified by DLS. Furthermore, the particles demonstrate considerable aggregation due to the lack of a capping agent, their relatively small size, and the natural inclination of IONPs to aggregate. Figure 9b depicts the TEM micrographs of the synthesized Fe_2_O_3_ NPs. The morphology of the nanoparticles ranged from 100 nm to 500 nm, and much larger agglomerated nanoparticles were observed^101^. To some extent, the reduction of the iron salts precursor was incomplete or inefficient and the addition of the NaBH_4_ reducing agent accomplish the rest iron salt traces.

Figure 9c represents TEM micrographs of the Ag-doped Fe_3_O_4_ NPs at different magnifications. Although both magnetic nanoparticles were polycrystalline, as revealed from the SAED pattern, they had an identifiable spherical shape and some noticeable differences. The TEM images revealed that the Ag-doped Fe_3_O_4_ NPs appeared darker and more compact than the Fe_3_O_4_ nanoparticles, indicating a greater density. This suggests that coating with Ag had a positive effect on the density of the Ag-doped Fe_3_O_4_ NPs^45^.

Figure 9d shows micrographs of the Ag-doped Fe_2_O_3_ nanoparticles at various magnifications and the particle size was heterogeneous. Furthermore, the SAED pattern confirmed that the obtained NPs were polycrystalline in nature, which is similar to the findings of^102^. Additionally, TEM analysis is an important technique for providing evidence of the formation of Ag-doped Fe_2_O_3_ nanoparticles. SAED meauements are preferable over XRD for assessing the crystalline structure, crystalline defects, and crystalline lattice parameters because XRD takes several centimeters of area, whereas SAED analyses several hundred nanometers in size. The substrate’s atoms serve as a grating to cause the diffraction of falling electrons, resulting in the appearance of a bright spot in the diffraction pattern.

#### DLS and zeta potential analysis

**Fig. 10:**
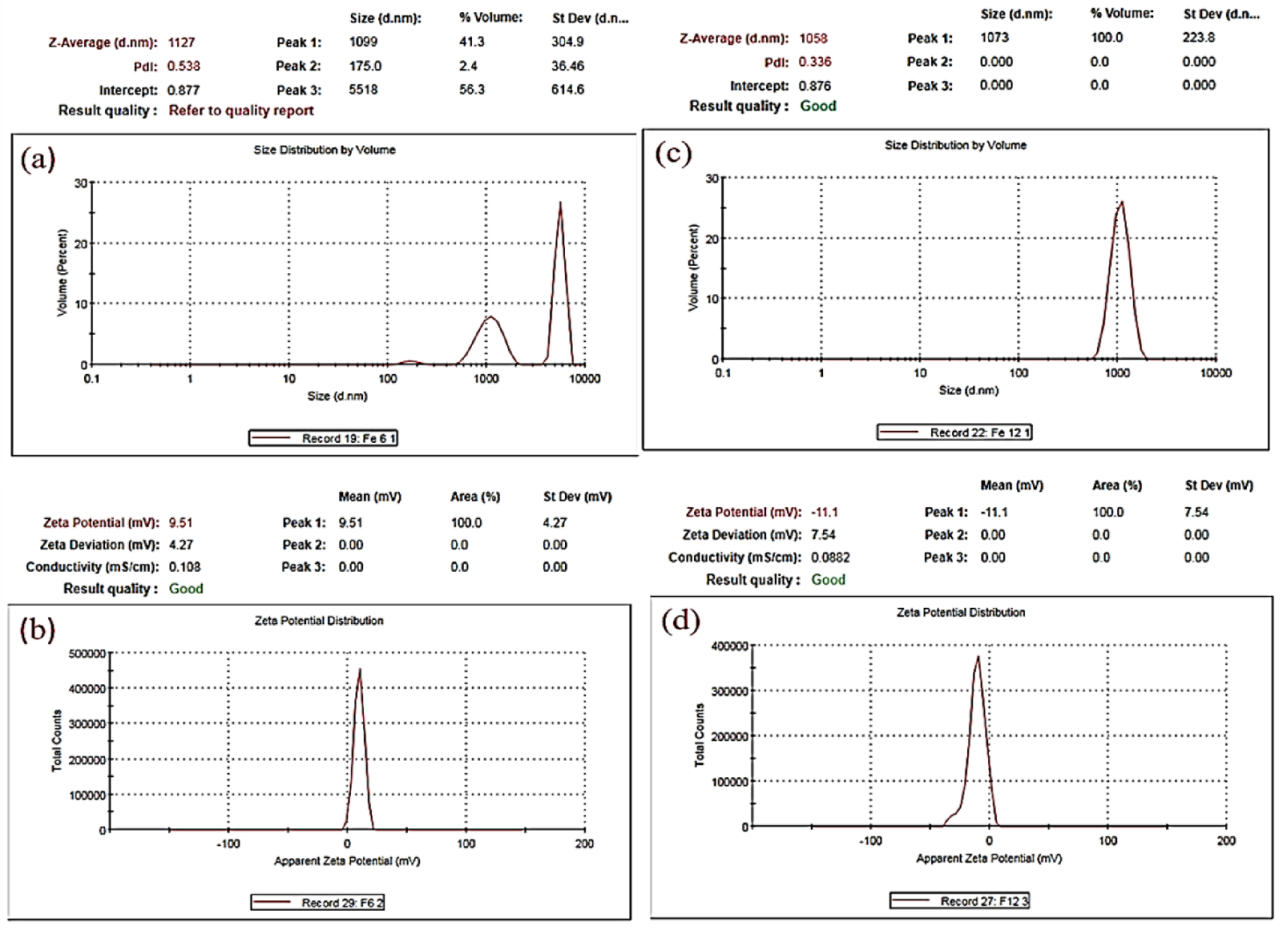
DLS and zeta potential of the synthesized IONPs. **(a)** DLS of Fe_3_O_4_ NPs, **(b)** zeta potential of Fe_3_O_4_ NPs, **(c)** DLS of Fe_2_O_3_ NPs, and **(d)** zeta potential of Fe_2_O_3_ NPs.

Figure 10a shows that the hydrodynamic size of the Fe_3_O_4_ NPs was heterogeneous, with sizes between 175 and 5518 nm and a 9.51 mV zeta potential **(**Fig. 10b**)**. The DLS results in Fig. 10c revealed that the average hydrodynamic size of the Fe_2_O_3_ NPs in aqueous solution was 1073 nm. The zeta potential distribution of the Fe_2_O_3_ NPs was quite narrow, centred at − 11.1 mV (Fig. 10d**)**.

**Fig. 11:**
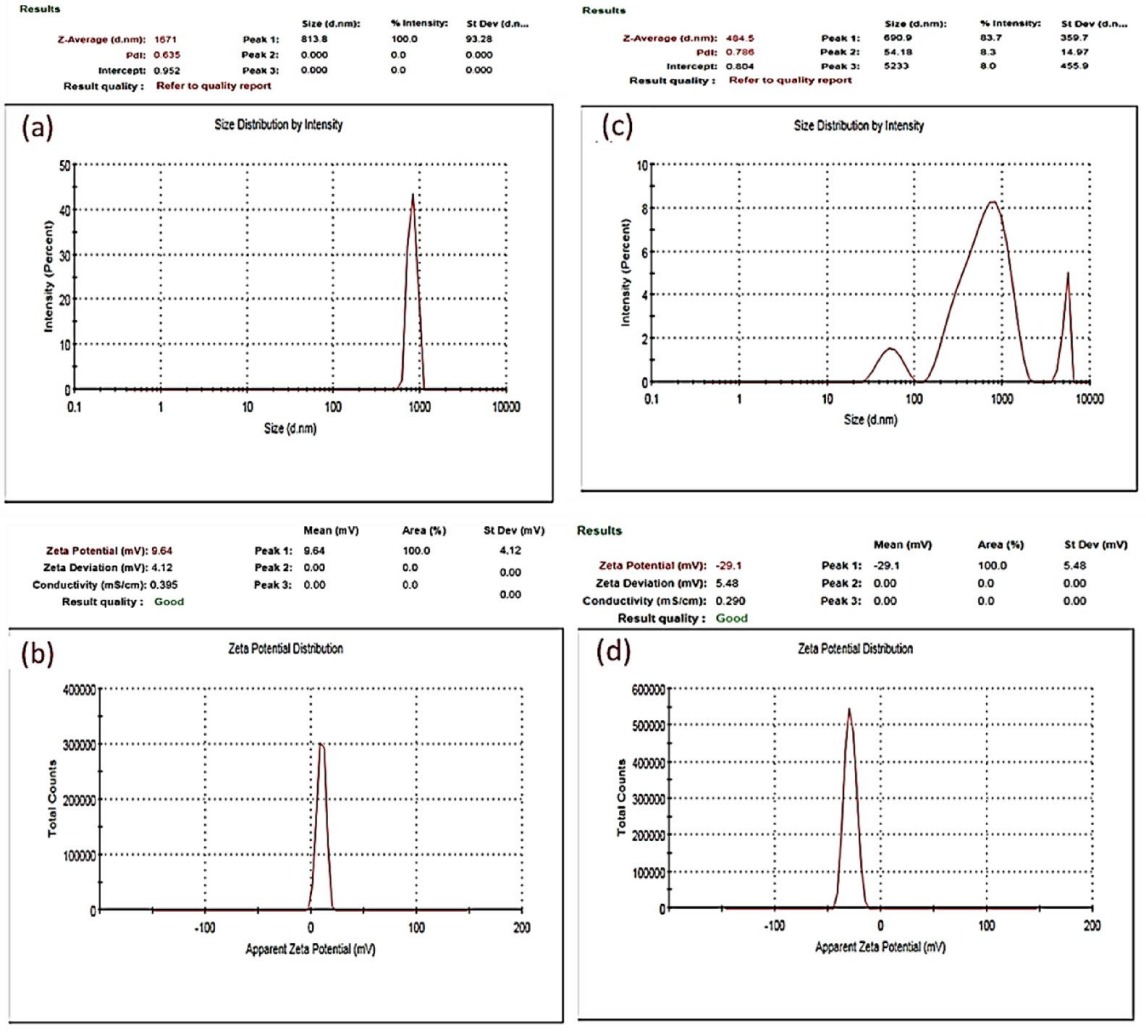
DLS and zeta potential of the synthesized silver-doped IONPs. **(a)** DLS of Ag-doped Fe_3_O_4_ NPs, **(b)** zeta potential of Ag-doped Fe_3_O_4_ NPs, **(c)** DLS of Ag-doped Fe_2_O_3_ NPs, and **(d)** zeta potential of Ag-doped Fe_2_O_3_ NPs.

Ag-doped Fe_3_O_4_ NPs and Ag-doped Fe_2_O_3_ NPs have hydrodynamic diameters of 813.8 nm and 54.18 to 5233 nm, respectively (Figs. 11a and c). These values are slightly greater than the results obtained from the TEM because the DLS measures the size of particles in solution and estimates the additional solvation shell of water; thus, the particles appear larger^103^. A zeta potential analyzer was also used to study the particle stability, with 9.64 and − 29.1 mV values for the Ag-doped Fe_3_O_4_ NPs and the Ag-doped Fe_2_O_3_ NPs, respectively (Figs. 11b and d). These results indicated that the stability of the IONPs improved after they were doped with silver.

### Biological evaluation of the synthesized ionps and silver-doped ionps

#### Antibacterial assay by well diffusion method

**Fig. 12:**
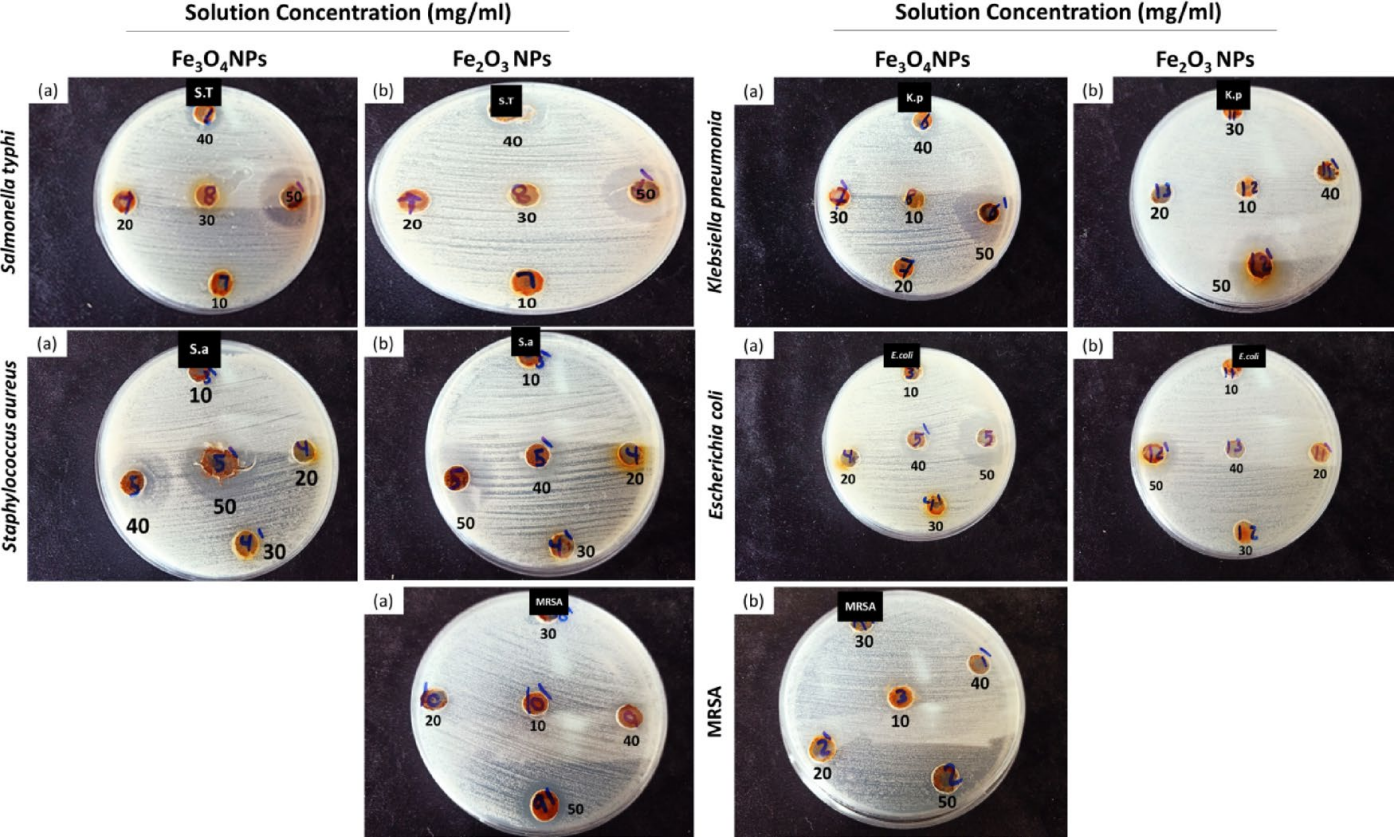
Representative plates showing the antibacterial activity of IONPs. **(a)** Effects of various concentrations of Fe_3_O_4_ NPs and **(b)** Fe_2_O_3_ NPs against the tested pathogenic bacteria.

By measuring microbial growth at specific concentrations of the tested material, the well diffusion assay was used to assess the antibacterial potency of the biosynthesized IONPs (Fe_3_O_4_ and Fe_2_O_3_ NPs). The results of the well diffusion test for nano-iron against *S. aureus*, *E. coli*, *K. pneumonia*, MRSA, and *S. typhi* are shown in Fig. 12. These results indicate the strong antimicrobial activity of IONPs against the Gram-negative bacterial strain *S. typhi* from 30 mg/ml in both the Fe_3_O_4_ and Fe_2_O_3_ NP samples. Additionally, the Gram-positive bacterial strain MRSA presented weak sensitivity after being treated with 50 mg/ml from Fe_3_O_4_. In contrast, the Fe_2_O_3_ NPs had no activity against it, as depicted in Table 1. This may be due to the presence of a thicker peptidoglycan layer^104^, where the presence of a thick peptidoglycan layer as an outer membrane functions as a permeability barrier that can also be vulnerable to the antibacterial effect of nanoparticles^105^. **Muthukumar**,** Chandrasekaran**^106^ reported a strong antimicrobial propensity of IONPs against Gram-negative bacterial strains. Both Gram-negative and Gram-positive bacteria were more sensitive to Fe_3_O_4_ than to Fe_2_O_3_. This may be attributed to the positive zeta potential of synthesized IONPs promotes their interactions through cell membranes and damages proteins, inner membranes, and DNA, reducing their viability^107^.

**Fig. 13:**
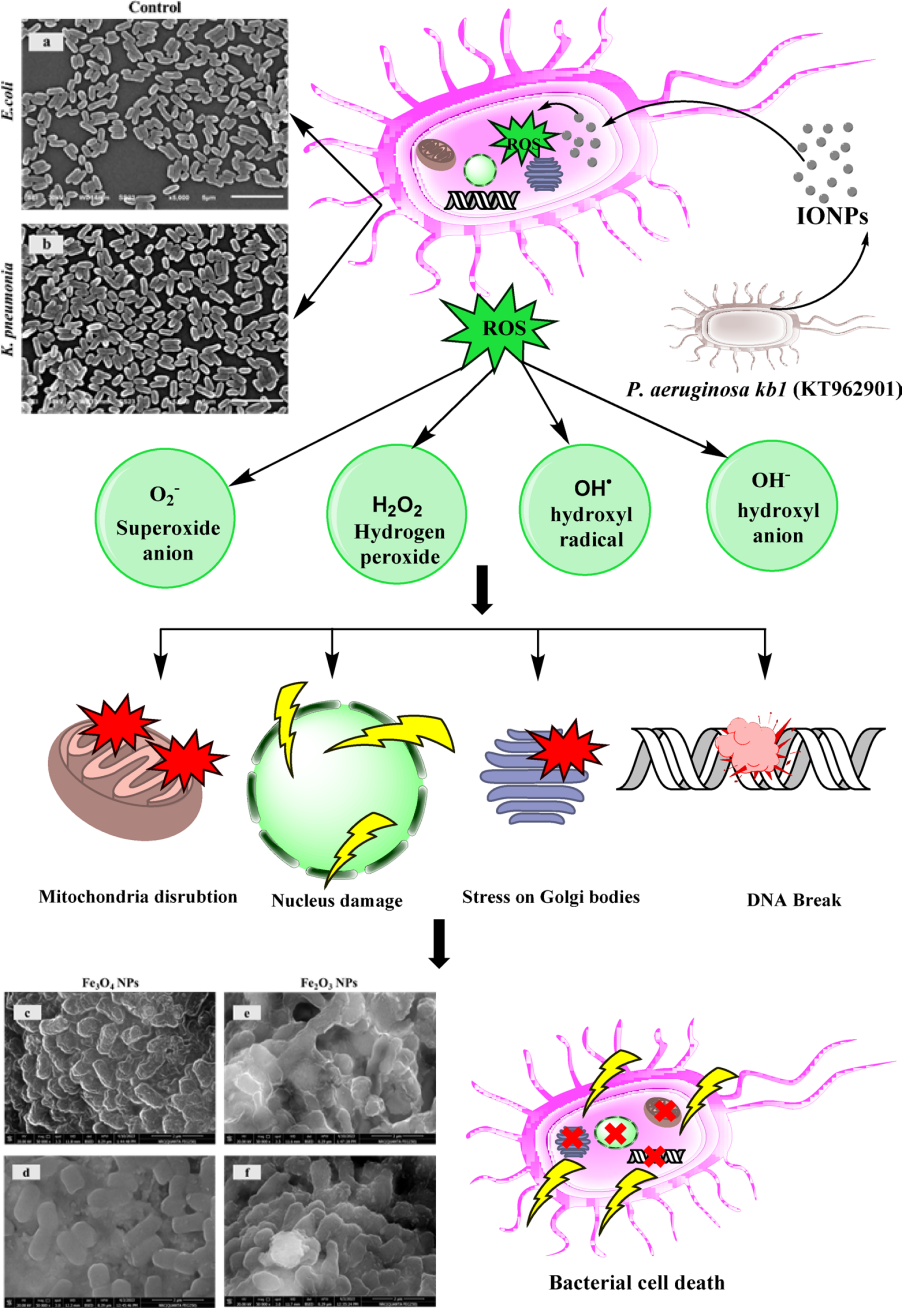
The proposed mechanism of the antibacterial activity of IONPs against the bacterial cells.

The main mechanism by which these metallic NPs show antibacterial activity might involve oxidative stress generated by reactive oxygen species (ROS)^108,109^, including superoxide radicals (O^2–^), hydroxyl radicals (-OH·), hydrogen peroxide (H_2_O_2_), and singlet oxygen (O_2_·), which can damage proteins and DNA in bacteria, as shown in Fig. 13^110^. However, there is variation in the inhibition of bacterial growth because of differences in several aspects, such as crystallite structure, surface morphology, particle size, and shape^111–113^.

**Fig. 14:**
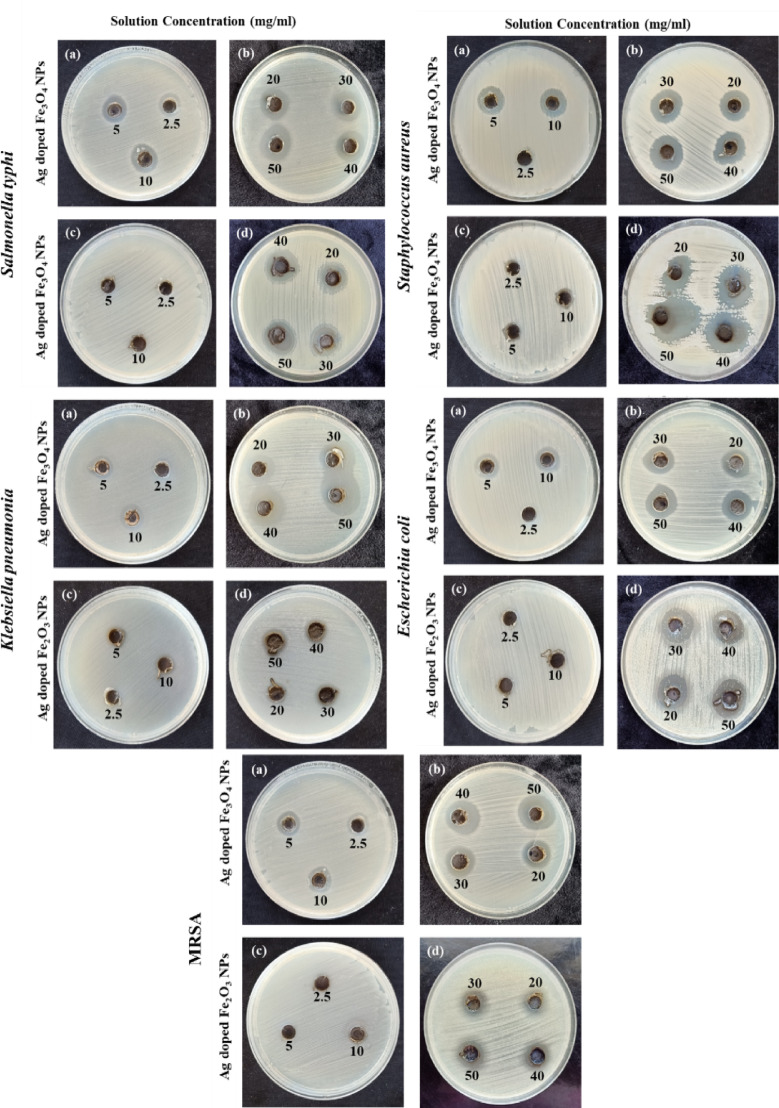
Representative plates showing the antibacterial activity of silver-doped iron NPs. **(a**,** b)** Ag-doped Fe_3_O_4_ NPs and **(c**,** d)** Ag-doped Fe_2_O_3_ NPs at various concentrations against the tested pathogenic bacteria.

The silver-doped ONPs exhibited a maximum inhibition zone of 23 mm for Ag-doped Fe_2_O_3_ nanoparticles against *S. aureus* as represented in Table 1. Conversely, the minimum inhibition zone recorded was 1.0 mm for Ag-doped Fe_3_O_4_ NPs at a concentration of 2.5 mg/ml against *K. pneumoniae* and *S. aureus*, as well as for Ag-doped Fe_2_O_3_ NPs at a concentration of 5 mg/ml against *K. pneumoniae*, as shown in Fig. 14.

Generally, the results obtained with silver-doped iron NPs were better than the results obtained with IONPs. Silver ions (Ag^+^) bind to the cell wall and cytoplasmic membrane, resulting in surface charge neutralization and permeability modification of the bacterial membrane^114^. Immediately after the import of free Ag^+^ ions into cells, respiratory enzymes can be inhibited, triggering reactive oxygen species that disrupt adenosine triphosphate formation and function as a main trigger for cell membrane breakdown and DNA alteration. Finally, it causes severe harm to bacterial cells^115–117^.

**Table 1:** The Inhibition (clear zone) values of ionps and silver-doped ionps with different concentrations against the tested bacterial isolates.

	Zone of growth inhibition in mm (Mean ± SE)					
	Concentration(mg/ml)	Bacterial Type				
		E.coli	k. pneumonia	S. aureus	S. typhi	MRSA
**Fe**_**3**_**O**_**4**_ **NPs**	**2.5**	N.D	N.D	N.D	N.D	N.D
	**5**	N.D	N.D	N.D	N.D	N.D
	**10**	N.D	N.D	N.D	N.D	N.D
	**20**	N.D	N.D	N.D	N.D	N.D
	**30**	N.D	N.D	N.D	4 ± 0.1	N.D
	**40**	6 ± 0.5	4 ± 0.10	11 ± 0.5	6 ± 0.25	N.D
	**50**	11 ± 0.25	11 ± 0.25	13 ± 0.1	11 ± 0.25	6 ± 0.53
**Fe**_**2**_**O**_**3**_ **NPs**	**2.5**	N.D	N.D	N.D	N.D	N.D
	**5**	N.D	N.D	N.D	N.D	N.D
	**10**	N.D	N.D	N.D	N.D	N.D
	**20**	N.D	N.D	N.D	N.D	N.D
	**30**	N.D	N.D	N.D	1 ±	N.D
	**40**	N.D	N.D	6 ± 0.53	6 ± 0.5	N.D
	**50**	9 ± 0.53	11 ± 0.25	11 ± 0.5	12 ± 0.1	N.D
**Ag-doped Fe**_**3**_**O**_**4**_ **NPs**	**2.5**	N.D	1 ± 0.2	1 ± 0.35	4 ± 0.5	4 ± 0.35
	**5**	4 ± 0.25	4 ± 0.35	6 ± 0.25	6 ± 0.35	5 ± 0.5
	**10**	5 ± 0.1	5 ± 0.25	7 ± 0.1	9 ± 0.1	5 ± 0.25
	**20**	8 ± 0.5	8 ± 0.1	9 ± 0.5	10 ± 0.1	8 ± 0.1
	**30**	11 ± 0.1	14 ± 0.1	10 ± 0.25	11 ± 0.5	9 ± 0.35
	**40**	11 ± 0.25	16 ± 0.1	11 ± 0.5	11 ± 0.1	13 ± 0.5
	**50**	12 ± 0.35	18 ± 0.5	12 ± 0.1	13 ± 0.25	14 ± 0.1
**Ag-doped Fe**_**2**_**O**_**3**_ **NPs**	**2.5**	N.D	N.D	N.D	N.D	N.D
	**5**	N.D	1 ± 0.1	N.D	N.D	N.D
	**10**	N.D	2 ± 0.2	N.D	N.D	N.D
	**20**	10 ± 0.2	2 ± 0.25	10 ± 0.35	11 ± 0.2	8 ± 0.1
	**30**	11 ± 0.2	3 ± 0.1	15 ± 0.35	13 ± 0.2	9 ± 0.25
	**40**	15 ± 0.35	7 ± 0.2	18 ± 0.1	13 ± 0.25	11 ± 0.35
	**50**	17 ± 0.2	8 ± 0.1	23 ± 0.2	19 ± 0.2	12 ± 0.5
**Positive control (µg/ml)**	**2.5**	N.D	2 ± 0.1	N.D	1 ± 0.1	N.D
	**5**	5 ± 0.2	3 ± 0.5	2 ± 0.3	4 ± 0.1	2 ± 0.5
	**10**	8 ± 0.35	3 ± 0.1	7 ±0.2	6 ± 0.5	4 ± 0.35
	**20**	8 ± 0.2	5 ± 0.5	9 ± 0.1	9 ± 0.25	9 ± 0.5
	**30**	15 ± 0.25	9 ± 0.35	13 ± 0.1	10 ± 0.5	9 ±0.2
	**40**	17 ± 0.25	11 ± 0.1	19 ± 0.35	13 ± 0.5	10 ± 0.35
	**50**	22 ± 0.2	12 ± 0.35	22 ± 0.1	19 ± 0.2	13 ± 0.5

N.D: Not detected; this means no antibacterial activity.

#### Growth curves of *E. coli* and *K. pneumoniae*

**Fig. 15:**
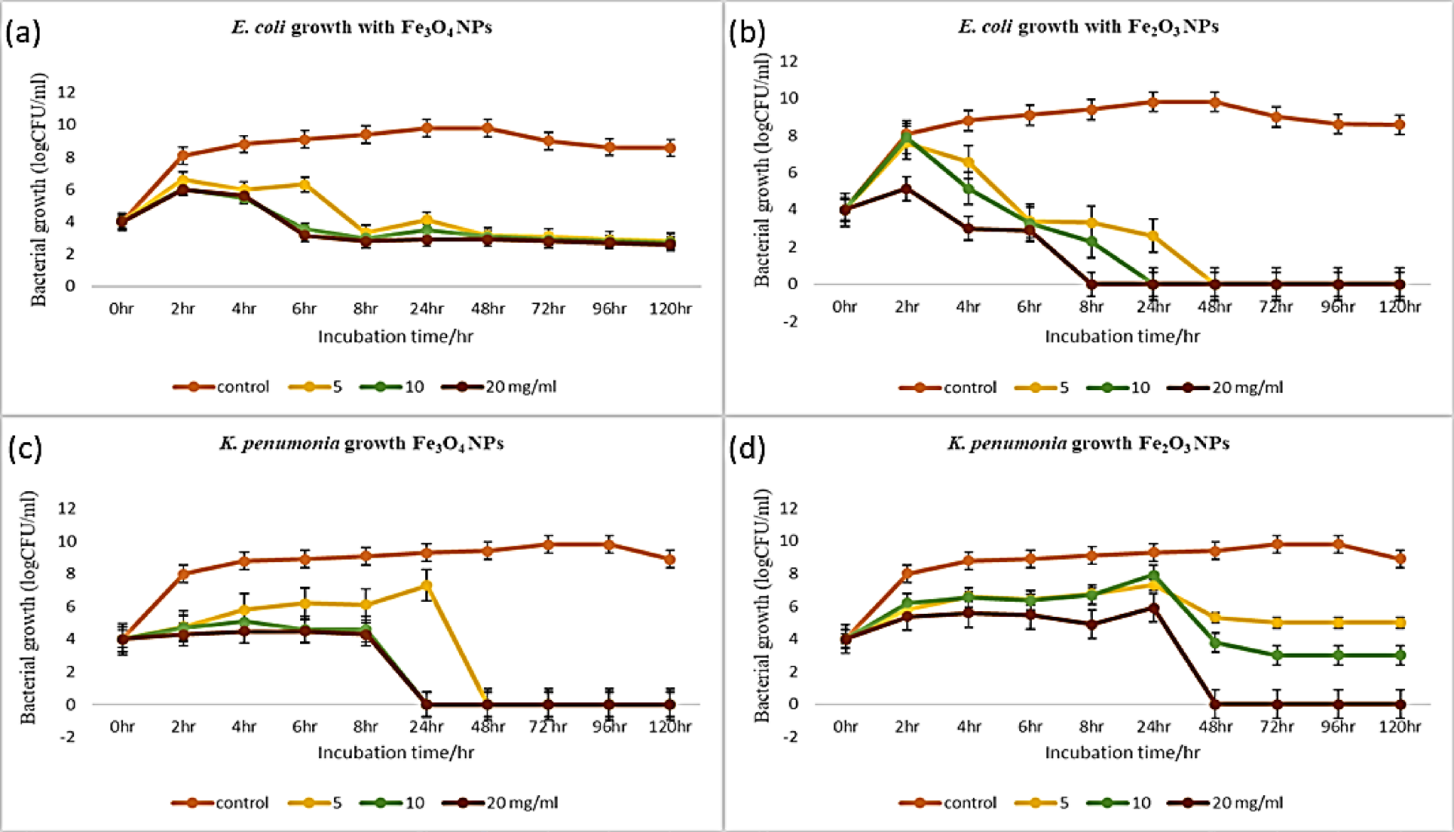
Growth curves of bacteria showing the effects of differently synthesized IONPs (Fe_3_O_4_ and Fe_2_O_3_) at different concentrations (5, 10, and 20 mg/ml) **(a)** *E. coli*, Fe_3_O_4_ NPs; **(b)** *E. coli*, Fe_2_O_3_ NPs **(c)** *K. pneumoniae*, Fe_3_O_4_ NPs **(d)** *K. pneumoniae*, Fe_2_O_3_ NPs.

The growth curves of *E. coli* and *K. pneumoniae* shown in Fig. 15 indicate that bacterial growth was inhibited by the addition of the biosynthesized IONPs (Fe_3_O_4_ and Fe_2_O_3_) compared with that of the control. Fe_3_O_4_ NPs were more effective against both *E. coli* and *K.* pneumoniae. In general, the higher the concentrations of IONPs applied against *E. coli* and *K. pneumoniae were*, the lower the growth values obtained. The growth of the control sample of *E. coli* increased gradually for 2 h, plateaued at 4 h, and then decreased after 48 h. Treating *E. coli* with 5, 10, and 20 mg/ml Fe_3_O_4_ and 20 mg/ml Fe_2_O_3_ decreased bacterial growth from the beginning of the incubation period compared with the normal growth of *E. coli*. In comparison, concentrations of 5 and 10 mg/ml Fe_2_O_3_ decreased *E. coli* growth after 2 h. The growth of the control sample of *K. pneumoniae* increased gradually for 2 h, plateaued at 4 h, and then decreased after 96 h. Fe_3_O_4_ NPs and Fe_2_O_3_ NPs at concentrations of 5, 10, and 20 mg/ml decreased the growth of *K. pneumoniae* immediately after the initial incubation period. **Muthukumar**,** Chandrasekaran**^106^ demonstrated the growth curve of *K. pneumoniae* in the presence of IONPs with no obvious growth after the fifth hour, whereas **Aiswarya Devi**,** Harshiny**^118^ reported that the growth curve of *E. coli* showed that the control rapidly reached the exponential phase. In contrast, in the presence of IONPs, the growth of *E. coli* is delayed.

**Fig. 16:**
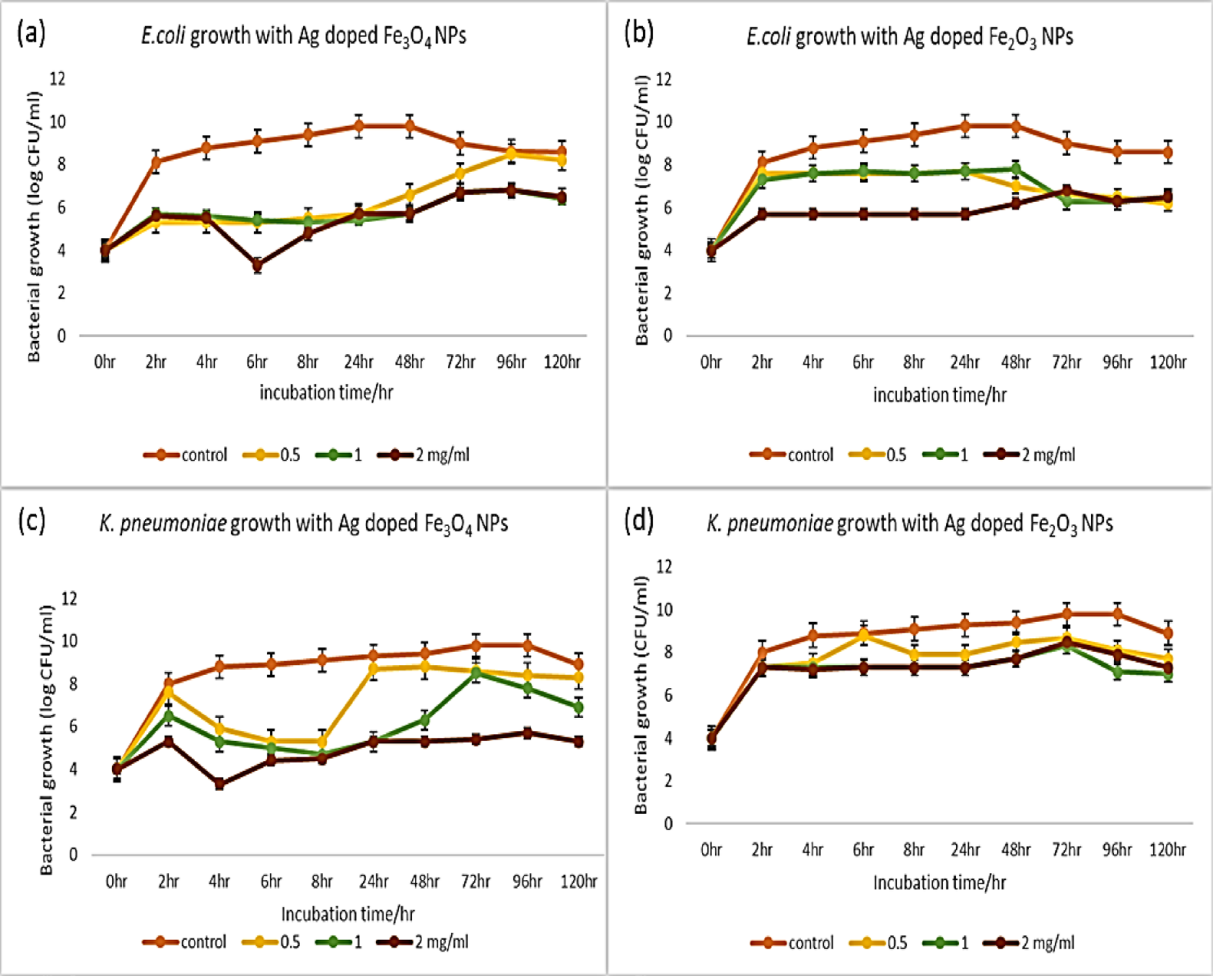
Growth curves of bacteria showing the effects of various concentrations of Ag-doped IONPs (0.5, 1, and 2 mg/ml). **(a)** *E. coli*, Ag-doped Fe_3_O_4_ NPs; **(b)** *E. coli*, Ag-doped Fe_2_O_3_ NPs; **(c)** *K. pneumoniae*, Ag-doped Fe_3_O_4_ NPs; **(d)** *K. pneumoniae*, Ag-doped Fe_2_O_3_ NPs.

In the case of silver-doped IONPs, Ag-doped Fe_3_O_4_ NPs were more effective against *E. coli* and *K. pneumonia*. Moreover, the higher the concentrations of Ag-doped iron NPs applied against both *E. coli* and *K. pneumoniae were*, the lower the growth values obtained. **Taylor**,** Kummer**^104^ proposed that the main reason for the antimicrobial activity of nanoparticles was the increase in the net interactive interaction at the nanobacterial interface. Above a threshold nanoparticle concentration, the interaction enhances ROS production at the interface^105^. Compared with the normal growth of *E. coli*,* treating E. coli* with Ag-doped Fe_3_O_4_ and 2 mg/ml Ag-doped Fe_2_O_3_ NPs decreased bacterial growth from the beginning of the incubation period. In comparison, 0.5 and 1 mg/ml Ag-doped Fe_2_O_3_ decreased *E. coli* growth after 2 h (Figs. 16a and b). Concentrations of 1 and 2 mg/ml Ag-doped Fe_3_O_4_ NPs decreased the growth of *K.* pneumoniae immediately from the beginning, whereas 0.5 mg/ml Ag-doped Fe_3_O_4_, as well as 1 and 2 mg/ml Ag-doped Fe_2_O_3_ NPs, reduced growth within 2 h, whereas 0.5 mg/ml Ag-doped Fe_2_O_3_ NPs started to inhibit bacterial growth within 6 h (Figs. 16c and d).

#### SEM analysis of *E. coli* and *K. pneumoniae*

**Fig. 17:**
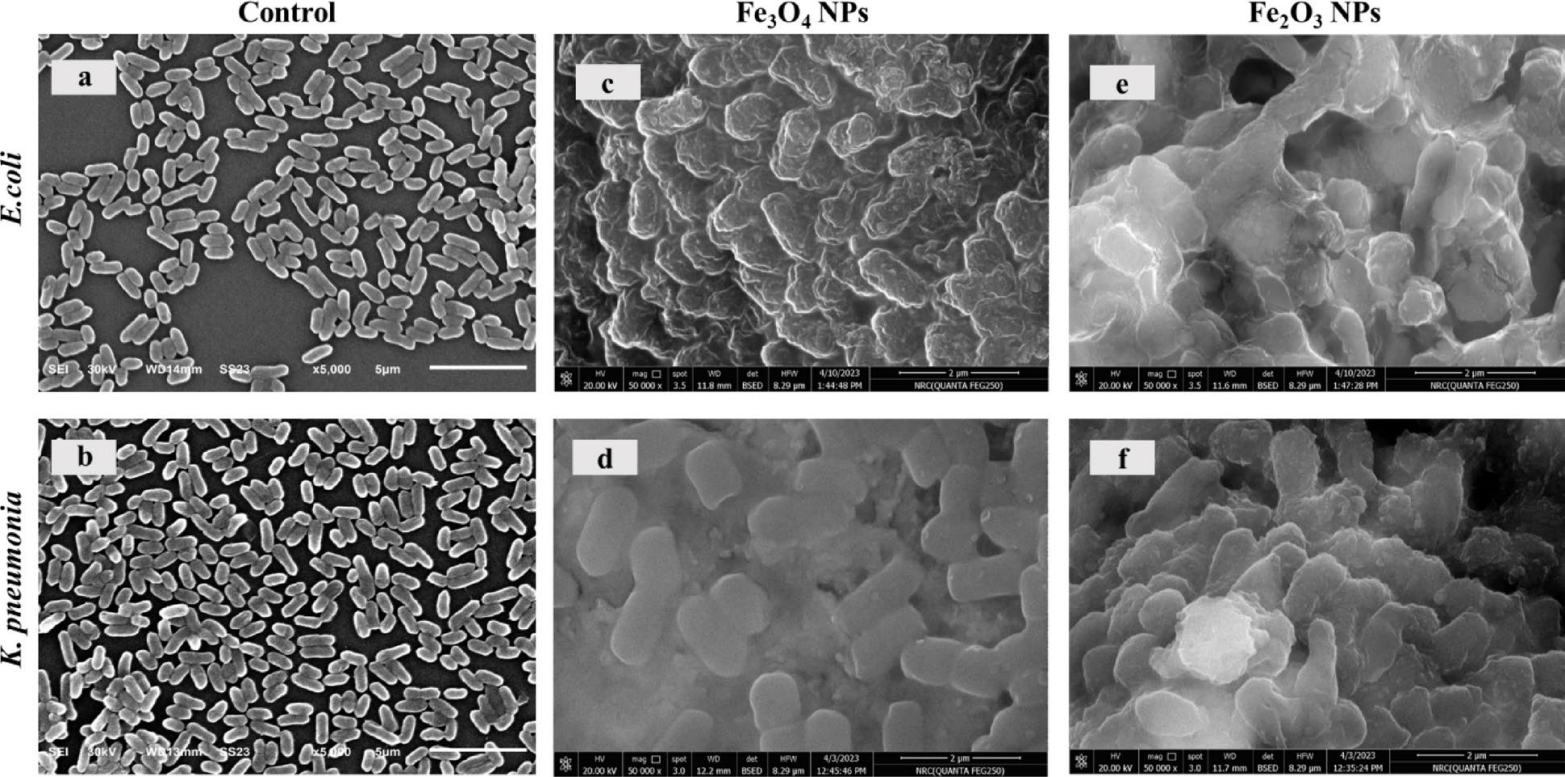
SEM micrographs of *E. coli* (**a**, **c**, and **e**) and *K. pneumoniae* (**b**, **d**, and **f**) before and after treatment with IONPs.

*E. coli* and *K. pneumoniae* were examined by scanning electron microscopy in the presence of IONPs based on the results of the dynamic growth curve of bacteria as represented in Fig. 17. The micrographs of the untreated *E. coli* and *K. pneumoniae* cells revealed the normal morphology of short rods and rod-shaped cells with intact and smooth cell walls as represented in Figs. 17a and b. On the other hand, after treatment with Fe_3_O_4_ and Fe_2_O_3_ NPs, most bacterial cells presented some changes in appearance. After the treatment of *E. coli* cells with both phases of IONPs, the cells appeared to be lysed after 24 h of incubation as illustrated in Figs. 17c and e. Additionally, the *K. pneumoniae* bacterial cell wall windings with abnormal streaks, leading to abnormal aggregation (Figs. 17d and f). These findings are inconsistent with the results obtained by those published by Irshad, R., et al.^119^.

#### Genotoxicity toward *E. coli* and *K. pneumoniae*

**Fig. 18:**
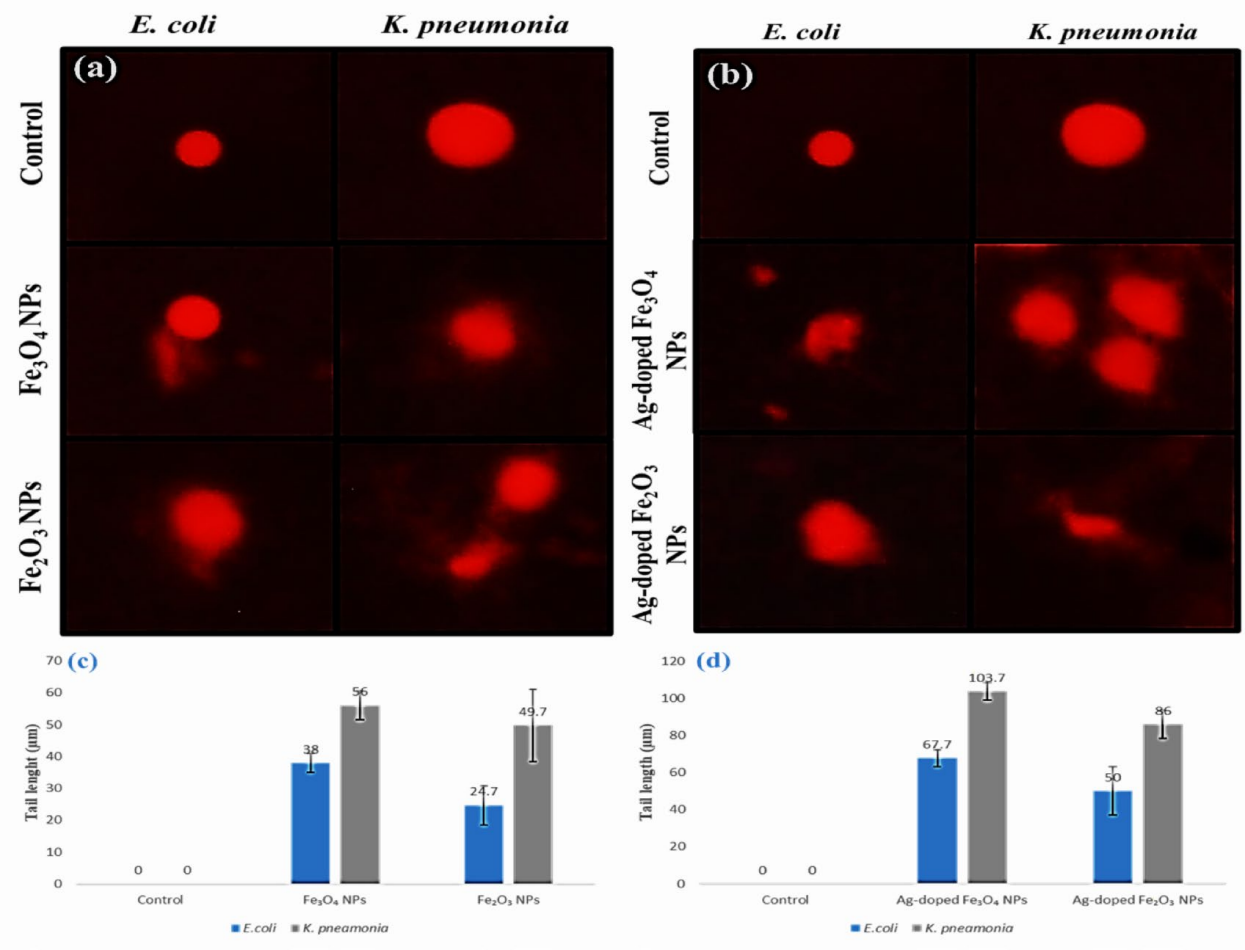
Representative images of comet assays of *E. coli* and *K. pneumoniae* before and after treatment with **(a)** Fe_3_O_4_ and Fe_2_O_3_ NPs and **(b)** Ag-doped Fe_3_O_4_ and Ag-doped Fe_2_O_3_ NPs. Bar diagram of the mean values of the tail length of *E. coli* and *K. pneumoniae* before and after treatment with **(c)** Fe_3_O_4_ and Fe_2_O_3_ NPs and (d) Ag-doped Fe_3_O_4_ and Ag-doped Fe_2_O_3_ NPs.

DNA damage was established as the length of the tail in the control and exposed cells. The data obtained from the comet assay after treatment of *E. coli* and *K. pneumoniae* with Fe_3_O_4_ and Fe_2_O_3_ NPs are shown in Figs. 18a and c. Compared with the control cells, the cells exposed to Fe_3_O_4_ and Fe_2_O_3_ NPs presented significantly greater DNA damage. This is a consequence of the reactive oxygen species (ROS) produced by IONPs in interaction with cytoplasmic proteins^120^. Even though low amounts of ROS are required to sustain the typical metabolism of cells, high levels of ROS can trigger DNA damage^121^. The tail length results indicated that bacterial DNA damage was more severe with Fe_3_O_4_ NPs than with Fe_2_O_3_ NPs in both *E. coli* and *K. pneumoniae*, and the genotoxicity of IONPs in response to either Fe_3_O_4_ or Fe_2_O_3_ NPs was greater in *K. pneumoniae* than in *E. coli*. Studies have shown that IONPs significantly induce DNA damage in BEAS-2B cells^122^. **Ahamed**,** A Alhadlaq**^123^ reported that relatively high concentrations of IONPs resulted in increased DNA damage in skin and lung epithelial cell lines. Despite the positive link between IONP consumption and genotoxicity indicated above, numerous additional investigations have revealed a negative relationship between IONP consumption and genotoxicity^124,125^.

Figures 18b and d revealed that the genotoxicity of Ag-doped IONPs to *E. coli* and *K. pneumoniae* was greater than that of IONPs. The degree of DNA damage caused by Ag-doped Fe_3_O_4_ NPs is greater than that caused by Ag-doped Fe_2_O_3_ NPs in both *E. coli* and *K. pneumoniae*, and the genotoxicity of Ag-doped IONPs, either Ag-doped Fe_3_O_4_ NPs or Ag-doped Fe_2_O_3_ NPs, was also greater in *K. pneumoniae* than in *E. coli*. This capacity may be drastically impacted by IONP properties, including size or surface coating type. NPs of different sizes may act differently and have varying genotoxic capabilities^126^. Silver NPs in both Ag-doped Fe_3_O_4_ and Ag-doped Fe_2_O_3_ NPs can form complexes with bases contained in DNA and are potent inhibitors of DNAase. Moreover, Ag can lead to enzyme inactivation via the formation of silver complexes with electron donors containing sulfur, oxygen, or nitrogen. The genotoxicity in *E. coli* and *K. pneumoniae* may be caused by the presence of sulfur and phosphorus as key components in DNA; silver particles can deal with these soft bases and break the DNA, resulting in cell death^127^. Other investigations employing the comet assay revealed that silver nanoparticles had favourable effects on human lung fibroblasts and human glioblastoma cells^120^.

#### Anticancer and cytotoxicity assay results

The viability of A549, HCT116, and RPE1 cells treated with the synthesized IONPs and Ag-doped IONPs decreased as the concentration increased. As an outcome, the findings demonstrated that the anticancer efficacy and cytotoxicity of synthesized nanoparticles are dependent on dose.

#### Lung cancer cell lines (A549)

**Fig. 19:**
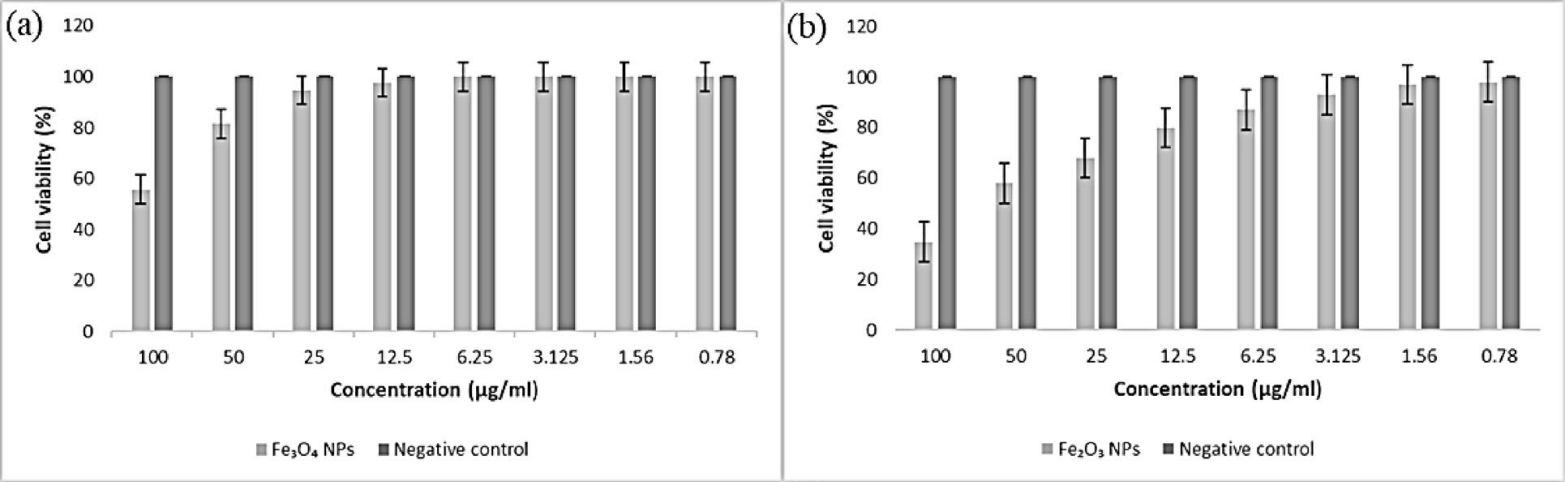
Bar diagram of in vitro anticancer studies of IONPs against a negative control lung carcinoma cell line. **(a)** Fe_3_O_4_ NPs, and **(b)** Fe_2_O_3_ NPs.

Figure 19 revealed that when the lung carcinoma cell line was exposed to various doses of the Fe_3_O_4_ NPs for 48 h, 44.2% of the cells lost viability at 100 ppm, which indicates that there is no significant anticancer activity of the Fe_3_O_4_ NPs at concentrations up to 100 ppm against the lung carcinoma cell lines. In contrast, the biosynthesized Fe_2_O_3_ NPs significantly inhibited the growth and proliferation of the lung carcinoma cell line at an IC_50_ of 69.4 µg/ml and an IC_90_ of 135.94 µg/ml, with 65.2% cell mortality at 100 ppm as depicted in Table 2. **Bagyalakshmi**,** Priyadarshini**^128^ reported that iron NPs derived from *Syzygium aromaticum* extract had a detected anticancer effect on A549 cancer cells. Furthermore, **Li**,** Chen**^129^ reported that iron magnetite nanoparticles might enhance the growth suppression of A549 cancer cells.

**Fig. 20:**
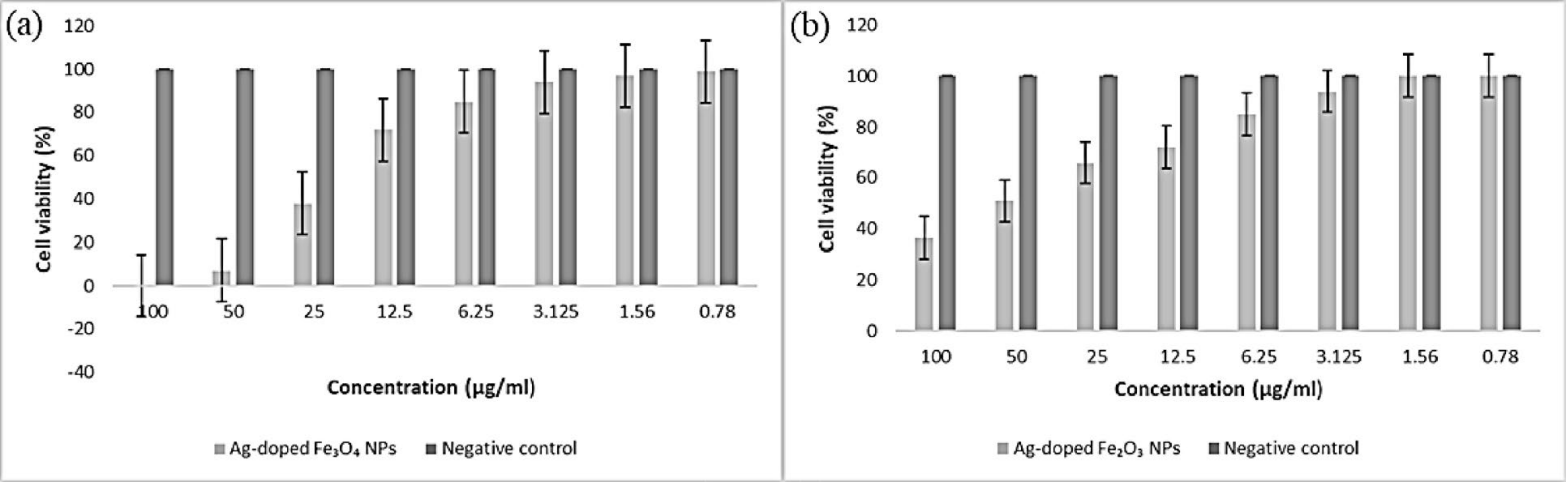
Bar diagram of in vitro anticancer studies of Ag-doped IONPs against a negative control lung carcinoma cell line. **(a)** Ag-doped Fe_3_O_4_ NPs, and **(b)** Ag-doped Fe_2_O_3_ NPs.

In the case of Ag-doped IONPs, both Ag-doped Fe_3_O_4_ NPs and Ag-doped Fe_2_O_3_ NPs exhibited remarkable anticancer activity in a lung cancer cell line, where Ag-doped Fe_3_O_4_ NPs were toxic, with an IC_50_ of 23.3 µg/ml and an IC_90_ of 41.5 µg/ml, with 100% cell mortality at 100 ppm as depicted in Table 3. In comparison, Ag-doped Fe_2_O_3_ NPs resulted in 63.5% cell mortality at 100 ppm, with an IC_50_ of 66.5 µg/ml and an IC_90_ of 134.0 µg/ml **(**Fig. 20**)**. The Ag-doped Fe_3_O_4_ NPs showed the maximum anticancer activity against the lung cancer cell line with the lowest IC_50_ value. However, the biosynthesized Fe_3_O_4_ NPs presented the lowest activity without silver doping.

Compared with undoped IONPs, Ag-doped IONPs had greater toxic effects on A549 cells. In addition, the IC_50_ of the Ag-doped Fe_3_O_4_ NPs was lower than that of doxorubicin (the control sample), so they can be considered a new group of drug-deliverable anticancer agents (Table 3). **Esmaeilzadeh**,** Rasoolzadegan**^130^ measured and reported the anticancer effects of Ag-doped IONPs on cancer cells.

**Table 2:** Results of the anticancer MTT assay of the effects of ionps on a lung carcinoma cell line (A549).

Tested treatment	IC_50_ (µg/ml)	IC_90_ (µg/ml)	Cell viability (%)
**Fe**_**3**_**O**_**4**_ **NPs**	N.D	N.D	55.8% at 100 ppm
**Fe**_**2**_**O**_**3**_ **NPs**	69.4	135.9	34.8% at 100 ppm
**DMSO**	N.D	N.D	95% at 100 ppm
**Negative control**	N.D	N.D	100%
**Positive control (Doxorubicin)**	28.3	46.5	0% at 100 ppm

N.D: Not detected; this means that the cell mortality is less than 50% at 100 ppm.

**Table 3:** Anticancer MTT assay results of Ag-doped ionps against a lung carcinoma cell line (A549).

Tested treatment	IC_50_ (µg/ml)	IC_90_ (µg/ml)	Cell viability (%)
**Ag-doped Fe**_**3**_**O**_**4**_ **NPs**	23.3	41.5	0% at 100 ppm
**Ag-doped Fe**_**2**_**O**_**3**_ **NPs**	66.5	134.0	36.5% at 100 ppm
**DMSO**	N.D	N.D	95% at 100 ppm
**Negative control**	N.D	N.D	100%
**Positive control (Doxorubicin)**	28.3	46.5	0% at 100 ppm

N.D: Not detected; this means that the cell mortality is less than 50% at 100 ppm.

#### Colon cancer cell line (HCT116)

**Fig. 21:**
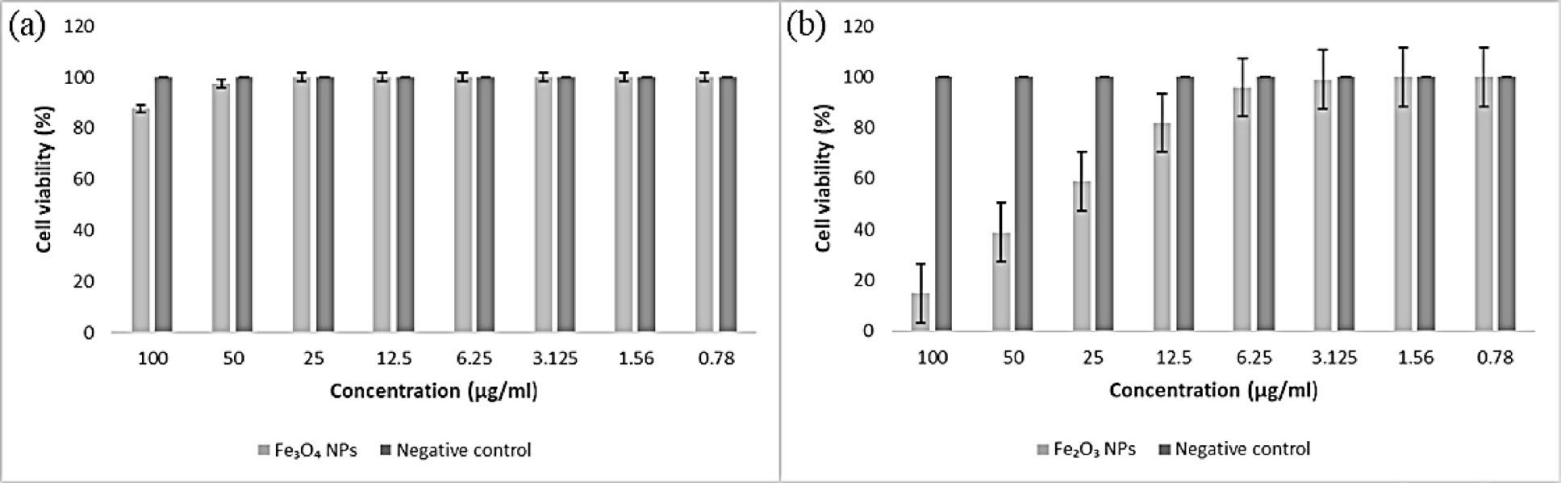
Bar diagram of in vitro anticancer studies of IONPs against a negative control colon cancer cell line. **a**: Fe_3_O_4_ NPs, **b**: Fe_2_O_3_ NPs.

Similar to the previous results, when the colon cancer cell line was exposed to several concentrations of the Fe_3_O_4_ and Fe_2_O_3_ NPs, the Fe_3_O_4_ NPs did not show notable anticancer activity, with 12.3% cell mortality at 100 ppm (Table 4**)**. In contrast, the Fe_2_O_3_ NPs suppressed the growth of colon cancer cells with 85.3% cell mortality at 100 ppm, with an IC_50_ of 50.4 µg/ml and an IC_90_ of 91.5 µg/ml, as shown in Fig. 21.

**Pillai**,** Sreelekshmi**^131^ Experiments demonstrated that the growth of human colon cancer cells was effectively suppressed via the biosynthesized IONPs. IONPs stimulate anticancer activity via both direct and indirect means through nontoxic wavelength radiation that is easily taken by toxic triggers of ROS generation. Furthermore, the nature of iron oxide particulates allows them to adhere covalently to tumour locations^132^.

**Table 4:** Results of the anticancer MTT assay of ionps against a colon cancer cell line (HCT116).

Tested treatment	IC_50_ (µg/ml)	IC_90_ (µg/ml)	Cell viability (%)
**Fe**_**3**_**O**_**4**_ **NPs**	N.D	N.D	87.7% at 100 ppm
**Fe**_**2**_**O**_**3**_ **NPs**	50.4	91.5	14.7% at 100 ppm
**DMSO**	N.D	N.D	99% at 100 ppm
**Negative control**	N.D	N.D	100%
**Positive control (Doxorubicin)**	37.6	42.6	0% at 100 ppm

N.D: Not detected; this means that the cell mortality is less than 50% at 100 ppm.

#### Normal retina cell line (RPE1)

**Fig. 22:**
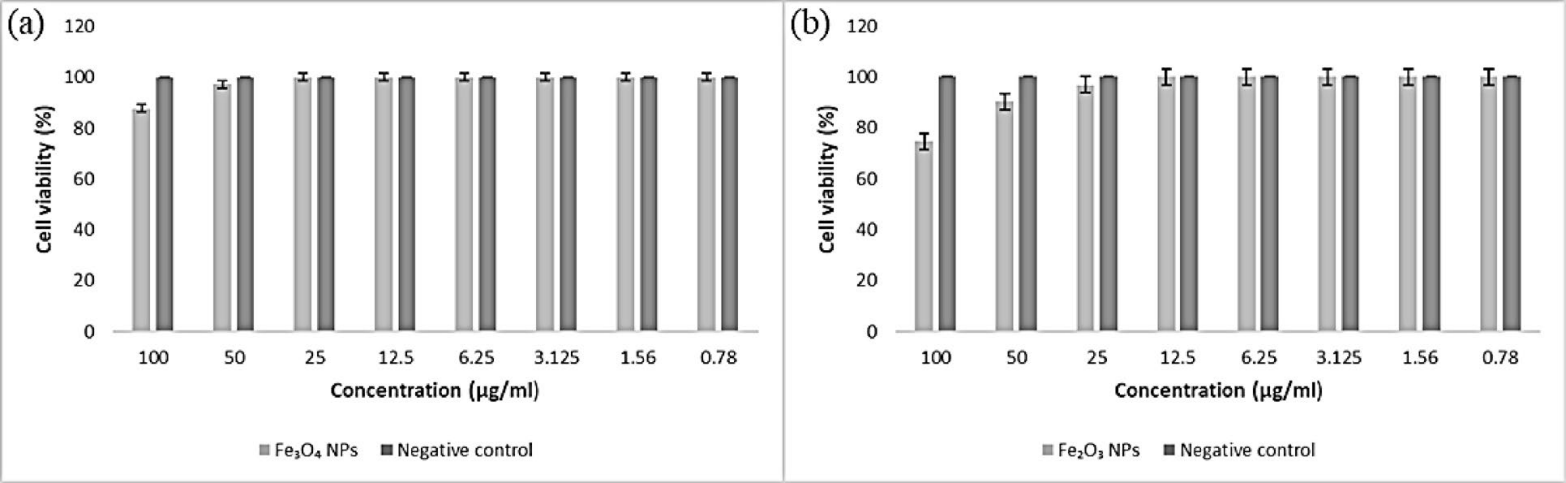
Bar diagram of in vitro anticancer studies of IONPs against the negative control normal retina cell line. **a**: Fe_3_O_4_ NPs, **b**: Fe_2_O_3_ NPs.

Figure 22 clearly shows that the green-synthesized IONPs, either Fe_3_O_4_ or Fe_2_O_3_ NPs, are considered nontoxic when exposed to normal cell lines, with 12.2% and 25.3% cell mortality at 100 ppm (Table 5), respectively.

**Fig. 23:**
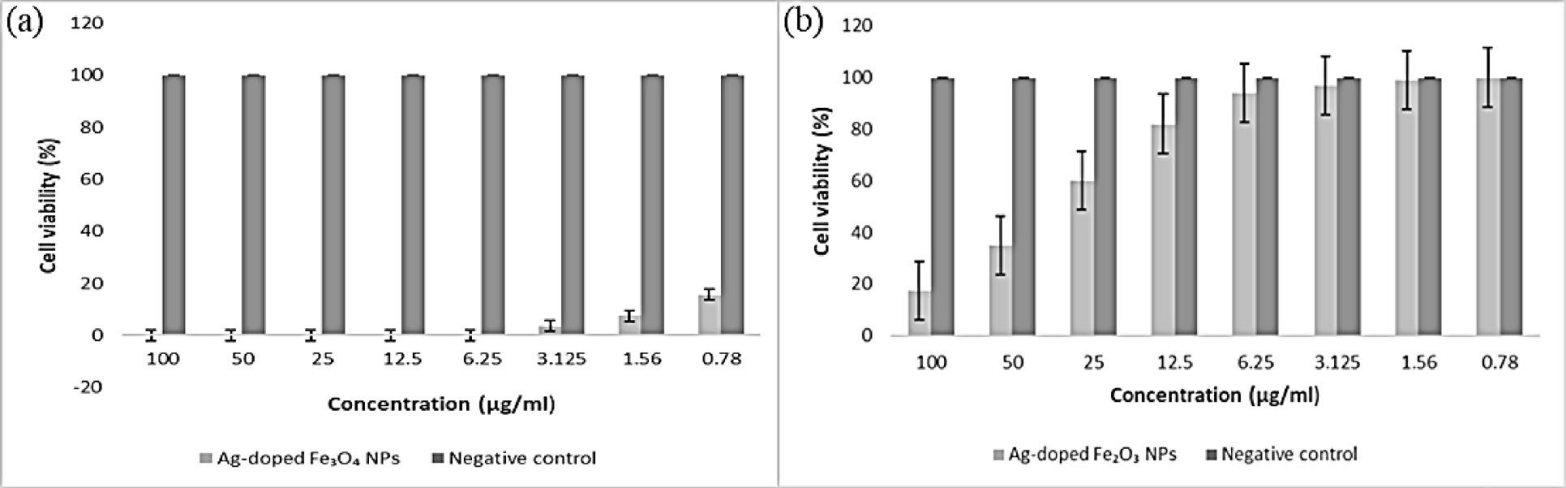
Bar diagram of in vitro anticancer studies of Ag-doped IONPs against the negative control normal retina cell line. **a**: Ag-doped Fe_3_O_4_ NPs, **b**: Ag-doped Fe_2_O_3_ NPs.

After the IONPs were doped with silver, the cytotoxic activity against the normal cell line increased to a maximum. The Ag-doped Fe_3_O_4_ NPs **(**Fig. 23**)** caused 100% cell mortality at concentrations of 6.25, 12.5, 25, 50, and 100 ppm, and the Ag-doped Fe_2_O_3_ NPs caused 82.5% cell mortality at 100 ppm, with an IC_50_ of 50.9 µg/ml and an IC_90_ of 94.7 µg/ml as depicted in Table 6. The toxicity of Ag-doped IONPs on the normal retina cell line is fairly clear, leading to their irreversible damage and death. This is comparable to the silver date of **Sarani**,** Hamidian**^133^.

**Table 5:** Cytotoxicity assay results of ionps against a normal retina cell line (RPE-1).

Tested treatment	IC_50_ (µg/ml)	IC_90_ (µg/ml)	Cell viability (%)
**Fe**_**3**_**O**_**4**_ **NPs**	N.D	N.D	87.8% at 100 ppm
**Fe**_**2**_**O**_**3**_ **NPs**	N.D	N.D	74.7% at 100 ppm
**DMSO**	N.D	N.D	95% at 100 ppm
**Negative control**	N.D	N.D	100%
**Positive control** **(Doxorubicin)**	13.5	30.1	0% at 100 ppm

N.D: Not detected; this means that the cell mortality is less than 50% at 100 ppm.

**Table 6:** Anticancer MTT assay results of Ag-doped ionps against a normal retina cell line (RPE1).

Tested treatment	IC_50_ (µg/ml)	IC_90_ (µg/ml)	Cell viability (%)
**Ag-doped Fe**_**3**_**O**_**4**_ **NPs**	N.D	1.3	0% at 6.25 ppm
**Ag-doped Fe**_**2**_**O**_**3**_ **NPs**	50.9	94.7	17.5% at 100 ppm
**DMSO**	N.D	N.D	95% at 100 ppm
**Negative control**	N.D	N.D	100%
**Positive control (Doxorubicin)**	13.5	30.1	0% at 100 ppm

N.D: Not detected; this means that the cell mortality is less than 50% at 100 ppm.

## Conclusion

Iron oxide nanoparticles (IONPs) have gained significant interest due to their unique anticancer, antioxidant, and antibacterial activities. The biosynthesis of IONPs was carried out by the magnetotactic bacterium *P. aeruginosa* kb1. To make Ag-doped IONPs, sodium borohydride (NaBH_4_) reduced the silver nitrate (AgNO_3_) salt on the previously prepared biosynthesized IONPs. SEM images showed that the nanoparticles clustered and had a uniform size distribution and approximately spherical shape. EDX and XRD analysis validated the production of maghemite (γ-Fe_2_O_3_) and magnetite (Fe_3_O_4_) IONPs. Fourier transform infrared spectroscopy determined the surface functional groups of Ag-doped and IONPs. Fe_3_O_4_ and Ag-doped Fe_3_O_4_ NPs had higher antibacterial activity against several harmful bacterial strains than Fe_2_O_3_ and Ag-doped Fe_2_O_3_. The normal retina and human lung cancer cell lines A549 were also tested for cytotoxicity using the MTT assay. Ag-doped Fe_3_O_4_ NPs were more cytotoxic than IONPs on A549 cells. However, Ag-doped Fe_3_O_4_ NPs did not harm normal retinal cell lines. In conclusion, according to the aforementioned obtained results, the eco-friendly biosynthesized Ag-doped Fe_3_O_4_ NPs, rather than other IONPs, are potentially preferable for biological and biomedical applications.

## Data Availability

This article contains all of the data created or analyzed during the present study.
